# Supplementary material for: New geographic model of care to manage the post-COVID-19 elective surgery aftershock in England: a retrospective observational study
Source: BMJ Open. 2020 Oct 31;10(10):e042392. doi: 10.1136/bmjopen-2020-042392 (PMC7783383; doi:10.1136/bmjopen-2020-042392)
Supplement: Supplementary data [file bmjopen-2020-042392supp001.pdf]

Appendix

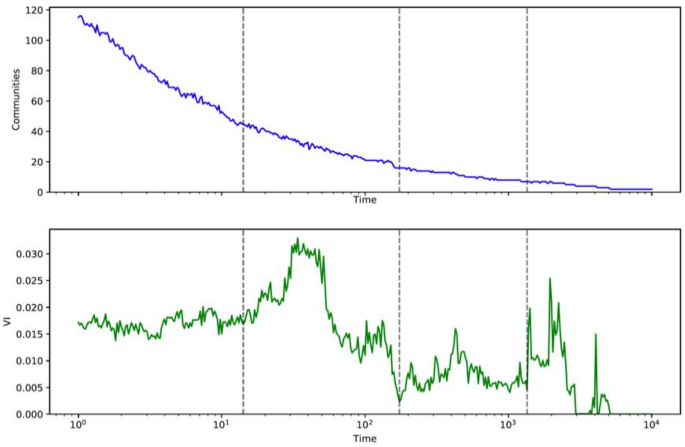

Supplemental Figure 1: Markov Multiscale Community Detection output showing the number of communities in the optimal network partition (top) and variation of information between partitions produced for Markov times from 1 to 10,000. Vertical lines indicate the three partitions of surgical communities selected for further review.
