# Supplementary material for: New geographic model of care to manage the post-COVID-19 elective surgery aftershock in England: a retrospective observational study
Source: BMJ Open. 2020 Oct 31;10(10):e042392. doi: 10.1136/bmjopen-2020-042392 (PMC7783383; doi:10.1136/bmjopen-2020-042392)
Supplement: Author's manuscript [file bmjopen-2020-042392.draft_revisions.pdf]

BMJ Open is committed to open peer review. As part of this commitment we make the peer review history of every article we publish publicly available.

When an article is published we post the peer reviewers' comments and the authors' responses online. We also post the versions of the paper that were used during peer review. These are the versions that the peer review comments apply to.

The versions of the paper that follow are the versions that were submitted during the peer review process. They are not the versions of record or the final published versions. They should not be cited or distributed as the published version of this manuscript.

BMJ Open is an open access journal and the full, final, typeset and author-corrected version of record of the manuscript is available on our site with no access controls, subscription charges or pay-per-view fees (http://bmjopen.bmj.com).

If you have any questions on BMJ Open's open peer review process please email info.bmjopen@bmj.com

A New Geographic Model of Care to Manage the Post-COVID-19 Elective Surgery Aftershock in England: A Retrospective Observational Study

Journal:	*BMJ Open*
Manuscript ID	bmjopen-2020-042392
Article Type:	Original research
Date Submitted by the Author:	06-Jul-2020
Complete List of Authors:	Clarke, Jonathan; Imperial College of Science Technology and Medicine, Department of Mathematics<br>Murray, Alice; Imperial College of Science Technology and Medicine, Department of Surgery and Cancer<br>Markar, Sheraz; Imperial College London, Department of Surgery and Cancer<br>Barahona, Mauricio; Imperial College of Science Technology and Medicine, Department of Mathematics<br>Kinross, James; Imperial College London, Division of Surgery
Keywords:	SURGERY, Health policy < HEALTH SERVICES ADMINISTRATION & MANAGEMENT, Organisation of health services < HEALTH SERVICES ADMINISTRATION & MANAGEMENT, PUBLIC HEALTH

SCHOLARONE™
Manuscripts

I, the Submitting Author has the right to grant and does grant on behalf of all authors of the Work (as defined in the below author licence), an exclusive licence and/or a non-exclusive licence for contributions from authors who are: i) UK Crown employees; ii) where BMJ has agreed a CC-BY licence shall apply, and/or iii) in accordance with the terms applicable for US Federal Government officers or employees acting as part of their official duties; on a worldwide, perpetual, irrevocable, royalty-free basis to BMJ Publishing Group Ltd ("BMJ") its licensees and where the relevant Journal is co-owned by BMJ to the co-owners of the Journal, to publish the Work in this journal and any other BMJ products and to exploit all rights, as set out in our licence.

The Submitting Author accepts and understands that any supply made under these terms is made by BMJ to the Submitting Author unless you are acting as an employee on behalf of your employer or a postgraduate student of an affiliated institution which is paying any applicable article publishing charge ("APC") for Open Access articles. Where the Submitting Author wishes to make the Work available on an Open Access basis (and intends to pay the relevant APC), the terms of reuse of such Open Access shall be governed by a Creative Commons licence – details of these licences and which Creative Commons licence will apply to this Work are set out in our licence referred to above.

Other than as permitted in any relevant BMJ Author's Self Archiving Policies, I confirm this Work has not been accepted for publication elsewhere, is not being considered for publication elsewhere and does not duplicate material already published. I confirm all authors consent to publication of this Work and authorise the granting of this licence.

For peer review only

A New Geographic Model of Care to Manage the Post-COVID-19 Elective Surgery Aftershock in England: A Retrospective Observational Study

Jonathan Clarke*, Alice Murray*, Sheraz Markar, Mauricio Barahona, James Kinross on behalf of the PanSurg Collaborative.

* These authors contributed equally to the study.

Jonathan M Clarke
Clinical Research Fellow, Department of Mathematics, Imperial College London, Huxley Building, South Kensington Campus, London, UK, SW7 2AZ
ORCID ID: 0000-0003-1495-7746

Alice Murray
General Surgical Registrar, Department of Surgery and Cancer, Imperial College London, London, UK, SW7 5NH
ORCID ID: 0000-0002-5058-0491

Sheraz Markar
NIHR Clinical Lecturer, Department of Surgery and Cancer, Imperial College London, London, UK, SW7 5NH

Mauricio Barahona
Professor of Mathematics, Department of Mathematics, Imperial College London, Huxley Building, South Kensington Campus, London, UK, SW7 2AZ
ORCID ID: 0000-0002-1089-5675

James Kinross
Senior Clinical Lecturer, Department of Surgery and Cancer, Imperial College London, London, UK, SW7 5NH

For peer review only

Correspondence to:

Jonathan Clarke

ORCID ID: 0000-0003-1495-7746

Department of Mathematics, Huxley Building, South Kensington Campus, London, SW7 2AZ, UK

j.clarke@imperial.ac.uk,

+44 (0) 7792 690037

Word Count: 3623

For peer review only

For peer review only - http://bmjopen.bmj.com/site/about/guidelines.xhtml

ABSTRACT

Objectives

The suspension of elective surgery during the COVID pandemic is unprecedented and has resulted in record volumes of patients waiting for operations. Novel approaches that maximise capacity and efficiency of surgical care are urgently required. This study applies Markov Multiscale Community Detection (MMCD), an unsupervised graph-based clustering framework, to identify new surgical care models based on pooled waiting lists delivered across an expanded network of surgical providers.

Design

Retrospective observational study using Hospital Episode Statistics.

Setting

Public and private hospitals providing surgical care to National Health Service (NHS) patients in England.

Participants

All adult patients resident in England undergoing NHS-funded planned surgical procedures between 1st April 2017 and 31st March 2018.

Main outcome measures

The identification of the most common planned surgical procedures in England (High Volume Procedures – HVP) and proportion of low, medium and high-risk patients undergoing each HVP. The mapping of hospitals providing surgical care onto optimised groupings based on patient usage data.

Results

A total of 7,811,891 planned operations were identified in 4,284,925 adults during the one-year period of our study. The 28 most common surgical procedures accounted for a combined 3,907,474 operations (50.0% of the total). 2,412,613 (61.7%) of these most

For peer review only - http://bmjopen.bmj.com/site/about/guidelines.xhtml

common procedures involved ‘low risk’ patients. Patients travelled an average of 11.3 km for these procedures. Based on the data, MMCD partitioned England into 45, 16 and 7 mutually exclusive and collectively exhaustive natural surgical communities of increasing coarseness. The coarser partitions into 16 and 7 surgical communities were shown to be associated with balanced supply and demand for surgical care within communities.

Conclusions

Pooled waiting lists for low risk elective procedures and patients across integrated, expanded natural surgical community networks have the potential to increase efficiency by innovatively flexing existing supply to better match demand.

For peer review only

Article Summary:

Strengths and Limitations of this Study

- The COVID-19 pandemic has significantly disrupted the provision of planned surgical care in hospitals across the world. Addressing the accumulated backlog of cases requires a new model of care whereby procedures are carried out at pace, while also responding to the dynamic risk of further COVID-19 outbreaks.
- This study finds that half of planned procedures in England are accounted for by only 28 types of procedure. Of these procedures 62% occur in low risk patients, and on average patients receive surgery only 11 km from their homes.
- We find that partitioning hospitals in England into 16 surgical communities balances local supply and demand for planned surgery, while allowing the hospitals to collaborate to share capacity.
- While this study advances the potential role of collaboration between surgical centres to address the surgical backlog resulting from COVID-19, it does not address issues relating to local financial or logistical barriers to implementation of such a strategy.

For peer review only - http://bmjopen.bmj.com/site/about/guidelines.xhtml

Introduction

The COVID-19 pandemic put a global halt to the majority of elective surgery in order to manage the surge in patients requiring acute hospital services and ITU care [1-4]. It has been estimated that 28 million elective operations worldwide have been cancelled or postponed due to the pandemic[5]. Although the focus of public health organisations globally was rightly mounting an effective emergency response to the COVID-19 pandemic, the surgical ‘aftershock’ will therefore be unprecedented and yet to be fully appreciated. Millions of patients in the UK are already waiting for treatment and numbers increase daily as the diversion of resources continues. [6] Elective surgical services are gradually being re-introduced, aiming to treat waiting patients without risking the spread of COVID. Management strategies in the UK are currently focused on undertaking life-saving cancer operations in “clean” COVID-free hospitals or in hospital sites away from the acute care sites where COVID is more prevalent [7] [8]. An immediate response to “catch up” and clear caseload will need to be undertaken, as well as adjusting to a “new normal”.

Waiting list numbers vary widely across the country and waiting times have increased in recent years[9]. To add complexity, there is also regional variation in the number of COVID infections and burden of COVID-related workload [10,11]. Therefore, in order to respond to the needs of a particular population, dynamic, flexible, regional solutions will be required to balance the reintroduction of services with careful COVID management.

Flexibility in the location where care is provided, according to patients’ clinical needs, has the potential to better match supply of services where there is appropriate demand. Patients can be treated more promptly if surgeons, hospitals and hospital delivery systems work together across provider networks, managing a centrally pooled workload. While some patients will need to be treated at specific locations (particularly high-risk patients or those requiring complex cancer care), there are other less complex procedures that could feasibly be performed by a range of qualified providers for patients who are able to travel.[12]

For peer review only - http://bmjopen.bmj.com/site/about/guidelines.xhtml

As the National Health Service (NHS) in England moves towards greater integration, there is an opportunity to break down arbitrary geographic boundaries and funding barriers, and bring together multiple providers of surgical care into 'surgical communities'. In such configurations, hospitals share a centrally managed waiting list for routine surgical procedures, and patients may receive surgery at any centre within the community of providers with the capacity to do so. There is a precedent for this approach, as a similar scheme was successfully piloted on a small scale in London [13]. Pooling available capacity between communities of surgical care providers may enable the efficient use of their collective available resources.

In this study we explore the potential of using flexible locations of care as a strategy to manage waiting lists. Firstly, we categorize the types of elective procedures and eligible patients into groups that would be amenable to undergoing surgery in any suitable location. Secondly, we identify from patient data existing community networks of surgical providers ('surgical communities') that collectively provide planned surgical care to similar geographic patient populations. Thirdly, we map these surgical communities against existing organizational configurations and model the effect on supply and demand when patients travel further for care.

Methods

All planned inpatient admissions to hospitals in England involving a surgical procedure were identified for adults resident in England from Hospital Episode Statistics from the 1st April 2017 to 31st March 2018. NHS-funded procedures conducted in non-NHS hospitals were included. For each admission, the first operative day was defined as the first day within an admission in which a surgical procedure was recorded. Procedures performed after the first operative day were excluded from the analysis.

All procedure codes describing diagnostic imaging, testing or rehabilitation (OPCS-4 codes beginning with U), the method of a procedure (Y) and site of a procedure (Z) were removed in addition to miscellaneous operations (X)[14]. Procedures involving the concurrent extraction of a lens (C71) and insertion of a prosthetic lens (C75) were treated

For peer review only - http://bmjopen.bmj.com/site/about/guidelines.xhtml

as a single procedure. Lower gastrointestinal diagnostic and therapeutic endoscopies frequently occurred concurrently or under codes with similar descriptions and were therefore grouped together. Conversely, diagnostic upper GI endoscopy (G45) was far more common than therapeutic endoscopies and was therefore treated separately.

Classification of Operative Risk

For each procedure, the age of the patient at the time of surgery was extracted. The modified Charlson comorbidity score of each patient was determined based on the presence of ICD-10 diagnosis codes extracted from their operative admission and all other recorded admissions to hospital for each patient in the 6 months prior to surgery[15]. Patients were then classified according to low, medium or high risk (for potential morbidity and mortality) by virtue of their age and Charlson Score (Table 1).

Identification of high-volume procedures

The total number of procedures performed for each 3-digit OPCS-4 code was calculated and sorted in descending order by volume. Those top procedures collectively accounting for more than 50% of the overall number of procedures were selected, and hereafter referred to as 'High Volume Procedures' (HVP).

Identification of hospital sites

The site in which a procedure was performed was identified from the SITETRET code of its associated admission. The postcodes of all sites in which procedures were performed were extracted from the site-level Estates Returns Information Collection.[16] Postcodes were converted to latitude and longitude coordinates. For all sites, the straight-line distance between all sites was calculated using the Haversine formula.[17] Where sites were within 1 km of one another, they were treated as a single merged site under the code and coordinates of the highest volume provider.

Calculation of Distance Travelled for Surgery

For each patient, the approximate location of their home was determined using the coordinates of the population-weighted centroid of their Lower Layer Super Output Area

For peer review only - http://bmjopen.bmj.com/site/about/guidelines.xhtml

(LSOA) of residence [18]. LSOAs are mutually exclusive, collectively exhaustive geographic census divisions defined by the UK Office for National Statistics, of which there are 32,844 in England, with a mean population of 1,704 people, and is therefore similar in scale to Census Block Groups in the Unites States. The straight-line distance between the population-weighted centroid of the LSOA of residence of the patient and the site in which the procedure was performed was calculated according to the Haversine formula.

For each HVP, the total number of procedures performed was calculated. The number of patients classified as low, medium and high risk was calculated, along with the total number of sites undertaking the procedure and the average distance travelled for surgery. For each HVP, the total number of procedures performed by each site was calculated. To exclude providers who rarely perform a procedure, the highest volume providers who collectively accounted for 99% of procedures were identified and classified as providers of the HVP.

Identification of Surgical Communities

The proportion of patients presenting from each LSOA in England to each Regular Provider site for a HVP was calculated and a normalised cosine similarity matrix of LSOAs was computed (Equation 1).

$$similarity_{AB} = \frac{\sum_{i=1}^{n} \boldsymbol{A_i B_i}}{\sqrt{\sum_{i=1}^{n} \boldsymbol{A_i^2}}\sqrt{\sum_{i=1}^{n} \boldsymbol{B_i^2}}} \qquad (1)$$

Equation 1: Calculation of cosine similarity between LSOAs. A_i is the proportion of patients presenting to hospital site i resident in LSOA A; B_i is the proportion of patients presenting to hospital site i resident in LSOA B; and n is the total number of hospital sites in the dataset.

This matrix quantifies the similarity of patterns of presentation for HVPs between all LSOAs in England. It can be understood as the adjacency matrix of a dense, weighted network connecting LSOAs to one another according to the similarity in their patterns of

For peer review only - http://bmjopen.bmj.com/site/about/guidelines.xhtml

presentation to hospital for HVPs.[19] This network was sparsened using the Relaxed Minimum Spanning Tree (RMST) technique, a method used elsewhere in applied network science to sparsen a dense, inhomogeneous network to preserve both local and global connectivity within a network. [20 21] This sparsened network was subsequently partitioned using Markov Multiscale Community Detection (MMCD) to produce partitions of the LSOAs according to shared patterns of presentation to hospital sites for HVPs. [22,23]

Description of Surgical Communities

The total number of procedures performed in each surgical community, and the total number of hospital sites was calculated. For each Sustainability and Transformation Partnership (STP - NHS organisational divisions of England into 44 regions responsible for developing local integration between primary and secondary care providers), the effective number of surgical communities active within its boundary was calculated using the Equivalent Market Size (the reciprocal of the Herfindahl Hirschman Index of market concentration) (Equation 2)[24]

$$EMS_i = 1/\sum_{j=1}^{N} s_{ij}^2 \qquad (2)$$

Equation 2: The Equivalent Market Size of of STP$_i$. Here s_{ij} is the proportion of LSOAs in STP i contained within surgical community j, and N is the number of surgical communities in the partition.

Calculation of the Balance Between Supply and Demand Within Surgical Communities

Surgical communities were modelled as self-contained subdivisions of England containing LSOAs contributing cases requiring surgery (demand) and hospitals providing finite surgical capacity for those services (supply).[25] In this configuration, surgical procedures for patients resident within a surgical community would be performed at a hospital site spatially located within the same surgical community. Within each surgical community, surgical demand was calculated as the total number of HVP cases performed for patients resident in LSOAs within the surgical community. Supply was calculated as

For peer review only - http://bmjopen.bmj.com/site/about/guidelines.xhtml

the total number of HVP cases performed by sites located within the geographic boundary of the surgical community. The supply-demand mismatch was calculated as the percentage difference between supply and demand for each community. The median of the absolute value of the supply-demand mismatch was determined.

Patient and Public Involvement

We did not directly include PPI in this study, but the database used in the study was released following review by a panel including patient representatives.

Results

A total of 7,811,891 planned interventional procedures corresponding to 5,718,031 admissions involving 4,284,925 adult patients resident in England from 1st April 2017 to 31st March 2018 were identified. There procedures were performed at 530 NHS hospital sites and 162 different private provider sites. 1,210 different 3-digit OPCS codes were used.

28 types of procedure in Table 2 accounted for 3,907,474 operations, over half of all planned surgical procedures during the study period. These are denoted as High Volume Procedures (HVPs). Of these HVPs, 3,553,649 (90.9%) were performed in an NHS site, while 353,825 (9.9%) were performed in a non-NHS site. Collectively, diagnostic or therapeutic upper and lower gastrointestinal endoscopy accounted for 1.6 million procedures (20.3%). On average, procedures were performed on patients aged 61.4 years (SD = 16.7 years). 2,636,559 procedures were performed on patients with a Charlson comorbidity score of 0 (67.5%), while 997,765 procedures were performed on patients with a Charlson score of 1 or 2 (25.5%) and 273,150 procedures were performed on patients with a Charlson score of 3 or more (7.0%).

The mean distance travelled from a patient's residence to hospital for surgery was on average 11.3 km. Mean distances for the 28 HVPs ranged from 9.4 km for upper GI endoscopy to 16.2 km for spinal nerve root injection. 2,412,613 (61.7%) HVPs were

For peer review only - http://bmjopen.bmj.com/site/about/guidelines.xhtml

performed in ‘low risk’ patients, 988,067 (25.3%) in ‘medium risk’ patients and 506,794 (13.0%) in ‘high risk’ patients. The proportion of procedures being performed on ‘high risk’ patients ranged from 1% for meniscal procedures to 52% for cystoscopy and resection of bladder lesions. In 22 out of 28 HVPs, more than 80% of patients were classified as ‘low’ or ‘medium’ risk.

Markov Multiscale Community Detection identified (see Figure 1b in the appendix) three robust community conformations of LSOAs consisting of 45 (Partition A), 16 (Partition B) and 7 (Partition C) surgical communities (Table 3 and Figure 1). Stable spatial motifs are observed across the three partitions.

Overlaid STP boundaries show variable agreement with surgical communities (Figure 1). Lower agreement is observed, for example in East Anglia, where surgical communities consistently partition in ‘north-south’ direction, while the STP boundary runs ‘east to west’. Close agreement can be seen in Cornwall, where STPs are adjoining, based around surgical communities. The Hampshire and Isle of Wight STP, in the south of England, remains divided between more than three surgical communities in Partition C.

The median number of HVP cases performed in each community ranges from 78,998 in the finest partition (A) to 574,403 in the coarsest partition (C). In Partition A, the median number of surgical sites per community is 9, with an interquartile range from 9-17. In Partition B the median number of surgical sites per community in Partition B is 25, with an interquartile range of 19-44, while in Partition C, a median of 84 surgical sites are present per community, with an interquartile range of 56 to 98. In Partition A, STPs involved a median of 1.7 surgical communities, compared to 1.1 for Partition B and 1.0 for Partition C. Only the Hampshire and the Isle of Wight STP remains divided between more than three surgical communities in Partition C (Figure 2).

Supply and Demand Relationships within Surgical Communities

In Partition A, median absolute percentage difference between supply and demand for HVPs within surgical communities is 5.1%. 12 communities (27%) had absolute

For peer review only - http://bmjopen.bmj.com/site/about/guidelines.xhtml

mismatches between supply and demand of more than 10%. These communities were located around conurbations in the North West of England and Greater London, with supply exceeding demand within cities, and demand exceeding supply in suburban communities. In Partition B, a supply demand mismatch exceeding 10% is only observed for the surgical community on the south of the Thames Estuary, where demand exceeds supply by 25%, indicating a role for nearby surgical sites in East London which lie outside of the community (Figure 3). In Partition C the percentage difference between supply and demand does not exceed 5% in any community.

For peer review only

Discussion

Hospital providers, policy makers and clinicians urgently require solutions for managing the COVID-19 elective surgical aftershock. This describes a state where COVID cases are in decline, in the context of strategically halted elective surgery and exponentially growing waiting lists. The extra-ordinary levels of demand for operations now requires radical new solutions to the way we organize and deliver surgical services. This study showed that there are existing hospital networks performing high volumes of low risk procedures for low risk, local patients. When we compare supply and demand for planned surgical care across England, the degree of mismatch varies widely, particularly around conurbations. Importantly, these data demonstrate that variation is reduced significantly when provider networks expand and smaller surgical communities coalesce into 16 larger geographic regions. We have identified a large group of potentially eligible, fit, lower risk patients who could be asked to travel greater distances than the existing median of 13 kms for their more minor surgery in order to shorten waiting times.

Central management of pooled waiting lists across an increased number of both NHS and non-NHS providers offers an opportunity for greater collaboration between surgical centres and a better distribution of workload. It would provide enhanced system resilience in the context of future COVID outbreaks to continue planned surgery in dedicated clean sites. [8,26,27] The scheme may have additional benefits including increased patient choice, greater workforce flexibility and maximization of teams across areas, with increasing efficiency. There is a paucity of high quality data on the effects of pooled waiting lists [28].

Some evidence for their potential success has come from smaller, single site initiatives piloting internal pooling of cases distributed to consultants in the same department. [29] [30] [30] Surgical pooling has been used successfully in crises to achieve waiting-list targets with work done by non-consultant grade surgeons and cases shifted to the private sector. Surgical pooling has also been successful in matching existing supply to demand across transplant networks where donors are matched to recipients across larger regions, and sometimes between countries.[31] Greater choice and increased competition between providers for patients can be associated with reduced waiting times [32].

The London Patient Choice Project (LPCP) was set up to reduce long waiting times for patients awaiting ophthalmic and minor general surgery procedures. Waiting lists were centrally pooled, managed and funded, with patients then given a choice on site of care in order to obtain earlier treatment. This lead to a convergence of waiting times across providers by relieving those hospitals with longer lists[33] [13] Central purchasing of services was likely key to its success. On the strength of this pilot project, the English NHS undertook a national roll-out of patient choice, but without the central purchasing or coordination. 'Choose & Book' offered patients a choice of at least four hospitals which led some patients to attend a hospital other than the nearest one. Unpicking the effect of Choose and Book on waiting lists separately to other initiatives piloted at the time is complex, but it is likely that the setting of targets and strong performance management were key drivers on reducing waiting times rather than patient choice alone. [34]

In the UK patients generally favour the convenience and familiarity of a local provider. However, a MORI poll for the BMA showed that if faced with a long wait, 27% of people would travel anywhere in the United Kingdom for treatment by the NHS[35]. 78% of patients surveyed in the Isle of Wight were willing to travel to the mainland for elective surgery where the wait was shorter [36]. Greater patient travel has the potential to alleviate focal strain on services, but it's practical application will require careful consideration. There are a number of barriers to travel – including patient mobility, age and risk as well as the cost of travel and the need for nearby family and friend support. In this study, selection of "low-risk patients and procedures" acts to mitigate some of these concerns, although the

For peer review only - http://bmjopen.bmj.com/site/about/guidelines.xhtml

identification of operative risk based on procedure, age and Charlson score may be limited, and clearly in practice a patient-specific, case-by-case approach would be required. Government subsidization of travel would be an important intervention to reduce inequalities based on socio-economic status, education level, vulnerability or social exclusion[35]. However, in the London pilot there was no evidence of inequalities in uptake of the pooled list scheme by social class, educational attainment, income or ethnicity[13]. In the UK, with increasing centralization of complex care, particularly cancer care, patients are often already asked to travel further.[37]

In this study we identified a degree of variation in the extent to which demand for planned surgery within a community is met by the capacity of hospitals located in the same community. This is in addition to the current variation in waiting list lengths and COVID infection and hospitalisation rates. If variability could be reduced, or eliminated, then capacity planning is simplified.[38] This strategy fits with NHS England's broader integration strategy as outlined in the Five Year Forward view and continued in the expansion of STPs to become larger integrated delivery systems.

There are a number of limitations to the study. The COVID-19 epidemic is without precedent in recent history, so it was not possible to make substantially data-driven assumptions. The government have previously advised reducing national travel as a public health tool to limit COVID spread [39]. While our model does encourage patient mobility and could be criticized for the risk of further spread, it also facilitates more effective regional strategies to dedicate sites as COVID clean or dirty. Stringent infection control measures will be an essential part of any reintroduction of routine services. Currently there is mounting evidence that patients are not seeking out routine care due to the perceived risk of COVID infection [40]. There is therefore a possibility that patients will choose not to undergo any elective procedures in the current climate, nor travel to an unknown hospital for that care. Pooled waiting lists are often disliked by surgeons who site the lack of autonomy and patient ownership with an increased risk of mis-diagnosis, unnecessary procedures listed, and unaddressed patient complexities [41,42]. These risks can, and should, be mitigated by ensuring clear standardised patient pathways, patient

For peer review only - http://bmjopen.bmj.com/site/about/guidelines.xhtml

triage and suitability assessments, clarity in the named responsible surgeon and pathways for ongoing continuity of care. Virtual platforms have become increasingly available during COVID allowing remote consultation and triaging of patients prior to any procedures[43]. Finally, while we have identified a mismatch between current policy (STP boundaries) and practice (the natural networks of surgical providers), we appreciate that implementation of new integrated networks on a larger scale would require significant new resources and planning. A new system of funding flows, mechanisms for regional waiting list coordination and a cost per case mechanism or other financial incentive would be required to support this new model.

The NHS, despite being centrally funded, functions as a disparate collection of separate providers with their own priorities and resource constraints. In the COVID-19 pandemic, pre-existing structures of service delivery within the NHS were temporarily transformed. Primary care providers collaborated at a regional level to provide COVID care through a network of hubs while hospitals collaborated with one another to ensure some cancer care could continue at a smaller number of 'clean' hospital sites. As health systems across the world look to address an ever-growing backlog for planned care created by COVID-19, this trend of enhanced collaboration must continue. If the NHS is to overcome this backlog and cope with further waves of COVID, providers of surgical care must develop the means by which they may share a collective caseload for low-risk patients. What is certain is that the NHS, along with most other healthcare delivery systems, is having to make seismic changes to the way it works in order to best manage ongoing complexities. This study provides a solution with greater regional capacity flexibility with which to respond and adapt. Re-designing arbitrary geographical boundaries to follow expanded natural surgical community networks has the potential to increase efficiency by flexing existing supply to meet demand. This, in addition to other key strategies, could have a profound effect on tackling the massive backlog of cases accruing during this deadly pandemic, thereby preventing further death, disability and reduced productivity from delayed surgery.

For peer review only - http://bmjopen.bmj.com/site/about/guidelines.xhtml

Author Contributions

JC and AM were involved in all aspects of the study. SM, MB and JK were involved in the conceptualization of the study and in the reviewing and editing of the draft. JC has had access to all the data in the study and all authors had final responsibility for the decision to submit for publication. JC attests that all listed authors meet authorship criteria and that no others meeting the criteria have been omitted.

Acknowledgements and Competing Interests

This article is independent research supported by grants from The Peter Sowerby Foundation and the National Institute for Health Research (NIHR) Imperial Patient Safety and Translational Research Centre (PSTRC) PSTRC_2016_004. Infrastructure support for this work was provided by the NIHR Imperial Biomedical Research Centre (BRC) 1215-20013. MB and JC acknowledge support from EPSRC grant EP/N014529/1 supporting the EPSRC Centre for Mathematics of Precision Healthcare. Johnson and Johnson have supported the activities of the Pansurg collaborative with an educational grant. Data management was provided by the Big Data and Analytical Unit (BDAU) at the Institute of Global Health Innovation (IGHI), Imperial College London. The views expressed in this publication are those of the author(s) and not necessarily those of the NHS, the National Institute for Health Research or the Department of Health.

Dissemination declaration

Data as to hospital allocation to putative surgical communities will be made publicly available on www.healthdatascience.co.uk and will be disseminated to local care providers and commissioners.

Data Sharing Statement

Data used in this study were obtained from NHS Digital for the purpose of this work and may only be accessed through direct application to NHS Digital. Patient-level data is required for the analyses conducted, and therefore sharing of data pertaining to this study

For peer review only - http://bmjopen.bmj.com/site/about/guidelines.xhtml

is not possible. Data for surgical community assignments are available from the authors on request.

Transparency declaration

The lead author affirms that this manuscript is an honest, accurate, and transparent account of the study being reported; that no important aspects of the study have been omitted; and that any discrepancies from the study as planned have been explained.

Statement of Ethical Approval

This study received local ethical approval through the Imperial College Research Ethics Committee (17IC4178).

For peer review only

For peer review only - http://bmjopen.bmj.com/site/about/guidelines.xhtml

References

1. K. Turaga SG. Are we harming cancer patients by delaying their cancer surgery during the COVID-19 pandemic? *Annals of Surgery* 2020.
2. Mary E Brindle GD, Keith Lillemoe, Atul Gawande. Approaching surgical triage during the COVID-19 Pandemic. *Annals of Surgery* 2020.
3. American College of Surgeons. COVID-19: Elective Case Triage Guidelines for Surgical Care. https://www.facs.org/covid-19/clinical-guidance/elective-case. Published 2020, March 24. Accessed.
4. Royal College of Surgeons of England. *COVID-19: Good Practice for Surgeons and Surgical Teams.* Royal College of Surgeons, England 2020.
5. Nepogodiev D, Bhangu A. Elective surgery cancellations due to the COVID-19 pandemic: global predictive modelling to inform surgical recovery plans. *The British journal of surgery.* 2020.
6. Brennan S. 'Mountain' waiting list will need five-year plan, says royal college. In. Vol 2020. Health Services Journal2020.
7. Brennan S. *Trusts told to create 'covid-free hubs' for cancer patients.* Health Services Journal 2020.
8. Kasivisvanathan V, Lindsay J, Rakhshani-moghadam S, et al. Evaluation of 30-day mortality for 500 patients undergoing non-emergency surgery in a COVID-19 cold site within a multicentre regional surgical network during the COVID-19 pandemic. *medRxiv.* 2020:2020.2006.2010.20115543.
9. National Audit Office. NHS waiting times for elective and cancer treatment. In: England DoHSCaN, ed2019.
10. House of Commons Committee of Public Accounts. *NHS waiting times for elective and cancer treatment.* Department of Health & Social Care and NHS England;March 2019.
11. Challen R T-AK, Pitt M et al. Estimates of regional infectivity of COVID-19 in the United Kingdom following imposition of social distancing measures. *medRxiv.* 2020.
12. Dixon A RR, Appleby J, Burge P, Devlin N, Magee H. *Patient Choice. How Patients Choose and How Providers Respond. .* The King's Fund;2010.
13. Dawson D, Gravelle H, Jacobs R, Martin S, Smith PC. The effects of expanding patient choice of provider on waiting times: evidence from a policy experiment. *Health Econ.* 2007;16(2):113-128.
14. Health and Social Care Information Centre. *National Clinical Coding Standards OPCS-4. Accurate data for quality information* 2019.
15. Quan H, Li B, Couris CM, et al. Updating and validating the Charlson comorbidity index and score for risk adjustment in hospital discharge abstracts using data from 6 countries. *Am J Epidemiol.* 2011;173(6):676-682.
16. NHS Digital. Estates Returns Information Collection. In: Collection S, ed. NHS Digital 2020.
17. Sinnott RW. Virtues of the Harversine *Sky and Telescope* 1984;68(2):158.
18. Office for National Statistics . Lower Layer Super Output Areas (December 2011) Population Weighted Centroids. https://geoportal.statistics.gov.uk/datasets/lower-

For peer review only - http://bmjopen.bmj.com/site/about/guidelines.xhtml

layer-super-output-areas-december-2011-population-weighted-centroids. Published 2019. Accessed.

19. Clarke JM, Barahona M, Darzi AW. Defining Hospital Catchment Areas Using Multiscale Community Detection: A Case Study for Planned Orthopaedic Care in England. *bioRxiv.* 2019:619692.
20. Beguerisse-Díaz M, Vangelov B, Barahona M. Finding role communities in directed networks using Role-Based Similarity, Markov Stability and the Relaxed Minimum Spanning Tree. Paper presented at: 2013 IEEE Global Conference on Signal and Information Processing; 3-5 Dec. 2013, 2013.
21. Altuncu MT, Mayer E, Yaliraki SN, Barahona M. From free text to clusters of content in health records: an unsupervised graph partitioning approach. *Applied Network Science.* 2019;4(1):2.
22. Schaub MT, Delvenne J-C, Yaliraki SN, Barahona M. Markov dynamics as a zooming lens for multiscale community detection: non clique-like communities and the field-of-view limit. *PloS one.* 2012;7(2):e32210-e32210.
23. Lambiotte R, Delvenne J, Barahona M. Random Walks, Markov Processes and the Multiscale Modular Organization of Complex Networks. *IEEE Transactions on Network Science and Engineering.* 2014;1(2):76-90.
24. Rhoades SA. Market share inequality, the HHI, and other measures of the firm-composition of a market. *Review of Industrial Organization.* 1995;10(6):657-674.
25. Martin S, Rice N, Jacobs R, Smith P. The market for elective surgery: joint estimation of supply and demand. *J Health Econ.* 2007;26(2):263-285.
26. NHS England. Advice on maintaining cancer treatment during the COVID-19 response. In: Improvement NEaN, ed2020.
27. PanSurg Collaborative. Exclusive: The three vital lessons Italian hospitals have learned in fighting covid-19. In: Journal HS, ed. Vol 2020. Health Services Journal2020.
28. Ballini L, Negro A, Maltoni S, et al. Interventions to reduce waiting times for elective procedures. *Cochrane Database of Systematic Reviews.* 2015(2).
29. Leach P, Rutherford SA, King AT, Leggate JR. Generic waiting lists for routine spinal surgery. *Journal of the Royal Society of Medicine.* 2004;97(3):119-120.
30. John Appleby AH, Nancy Devlin. *What is the Real Cost of More Patient Choice?* : The King's Fund;2003.
31. Madsen M, Asmundsson P, Brekke IB, et al. Scandiatransplant: thirty years of cooperation in organ transplantation in the Nordic countries. *Clin Transpl.* 1998:121-131.
32. Siciliani L, Martin S. An empirical analysis of the impact of choice on waiting times. *Health Econ.* 2007;16(8):763-779.
33. Dawson D, Jacobs R, Martin S, Smith P. Is patient choice an effective mechanism to reduce waiting times? *Appl Health Econ Health Policy.* 2004;3(4):195-203.
34. Dixon A. *New Labour's Market Reforms - Patient Choice of Hospital.* 2011.
35. Damiani M, Propper C, Dixon J. Mapping choice in the NHS: cross sectional study of routinely collected data. *BMJ (Clinical research ed).* 2005;330(7486):284.

For peer review only - http://bmjopen.bmj.com/site/about/guidelines.xhtml

36. Ryan M, McIntosh E, Dean T, Old P. Trade-offs between location and waiting times in the provision of health care: the case of elective surgery on the Isle of Wight. *J Public Health Med.* 2000;22(2):202-210.
37. Aggarwal A, Lewis D, Mason M, Purushotham A, Sullivan R, van der Meulen J. Effect of patient choice and hospital competition on service configuration and technology adoption within cancer surgery: a national, population-based study. *Lancet Oncol.* 2017;18(11):1445-1453.
38. Pandit JJ, Pandit M, Reynard JM. Understanding waiting lists as the matching of surgical capacity to demand: are we wasting enough surgical time? *Anaesthesia.* 2010;65(6):625-640.
39. The Health Protection Agency (Coronavirus, Restrictions) (England) Regulations 2020. In:2020.
40. Owens B. Reduced attendances and admissions: lessons from the covid crisis. In: Journal HS, ed. Vol 2020: HSJ; 2020.
41. Ramchandani M, Mirza S, Sharma A, Kirkby G. Pooled cataract waiting lists: views of hospital consultants, general practitioners and patients. *Journal of the Royal Society of Medicine.* 2002;95(12):598-600.
42. Position Statement on Pooled Waiting Lists [press release]. British Orthopaedic Association 2013.
43. NHS England. Elective Care Transformation Programme In: England N, ed2020.

For peer review only

Figure captions:

Figure 1: Division of England into 45, 16 and 7 surgical communities (in colour). according to Markov Stability. STP boundaries are overlaid (black lines).

Figure 2: The equivalent number of surgical communities active in each STP as determined by the EMS. Areas of darker blue, 4 (e.g. East Anglia), represent those areas with greatest difference between surgical communities and STPs, whereas lighter blue shows greater agreement (e.g. Cornwall).

Figure 3: The absolute percentage difference between the number of patients undergoing surgery resident within a surgical community (demand) and the number of procedures performed by hospitals within the community (supply). Areas in blue represent those surgical communities where procedures performed on patients outnumber those performed by the local providers.

For peer review only

For peer review only - http://bmjopen.bmj.com/site/about/guidelines.xhtml

		Charlson Score		
		0	1-2	3+
**Age**	< 60	Low	Low	Medium
	60 - 74	Low	Medium	High
	75+	Medium	High	High

Table 1: Classification of low, medium and high-risk patients based on age and Charlson score.

For peer review only

		Patient risk			
Procedure	Total no. of cases	Low risk (%)	Medium risk (%)	High risk (%)	Mean distance travelled, Kms
Lower GI endoscopy	937,616	74.8	17.9	7.3	9.8
Upper GI endoscopy	650,133	66.9	22.1	10.9	9.4
Lens extraction + replacement	395,445	33.5	46.5	20.0	10.9
Excision of skin lesion	215,608	55.0	29.5	15.5	12.7
Injection/aspiration joint	142,562	71.6	20.9	7.5	12.6
Vitrectomy	132,938	39.9	44.1	16.1	13.2
Cystoscopy	130,114	56.4	26.2	17.4	11.8
Insertion central venous catheter	109,864	24.3	38.3	37.4	14.0
Coronary angiography	105,620	56.2	30.0	13.8	13.9
Dental extraction	101,435	91.6	5.8	2.5	11.5
Knee replacement	78,773	53.3	34.4	12.3	13.4
Bladder catheterisation or irrigation	71,552	42.5	32.7	24.8	12.8
Injection to bladder	67,167	34.3	29.8	35.9	11.5
Spinal facet joint injection	64,154	70.4	21.9	7.7	14.0
Cholecystectomy	61,790	80.5	13.8	5.7	11.8
Lymph node biopsy	60,674	34.8	34.4	30.8	14.9
Epidural or spinal injection	60,656	69.2	22.6	8.1	12.9
Inguinal hernia repair	58,943	72.6	19.9	7.5	10.9
Spinal nerve root injection	58,212	77.0	17.5	5.5	16.2
Knee meniscectomy/ meniscal repair	57,871	93.2	5.9	0.8	12.4
Hysteroscopy	52,360	90.9	6.4	2.7	9.9
Carpal tunnel release	48,245	70.7	22.2	7.1	11.0
Application/ removal of internal fixation of bone	46,771	84.6	11.9	3.5	15.7
Dental clearance	43,463	82.3	11.7	5.9	11.1
Partial breast excision	41,827	50.1	31.4	18.5	11.5
Bone marrow biopsy	38,369	39.8	34.8	25.5	15.6
Primary joint resurfacing	37,854	59.3	30.1	10.5	14.0
Cystoscopy + resection of bladder lesion	37,458	17.7	29.8	52.5	11.2

Table 2: The 28 procedures accounting for more than half of all elective surgical activity in England. The proportion of patients classified as low, medium and high risk according to Table 1 are shown, along with the mean distance travelled from a patient's LSOA of residence to the hospital site in which the procedure is performed.

For peer review only - http://bmjopen.bmj.com/site/about/guidelines.xhtml

Partition	A	B	C
Number of communities	45	16	7
Median number of cases per community	78998 (43628 - 118087)	214216 (122823 - 314022)	574403 (406465 - 679703)
Median number of treatment sites per community	9 (5 - 17)	25 (19 - 44)	84 (56 - 98)
Absolute supply:demand mismatch (%)	5.1 (2.9 - 10.2)	4.1 (1.0 - 5.7)	2.2 (1.0 - 2.9)

Table 3: Descriptive statistics for the three optimal partitions produced. Interquartile ranges are shown in parentheses where appropriate.

For peer review only

For peer review only - http://bmjopen.bmj.com/site/about/guidelines.xhtml

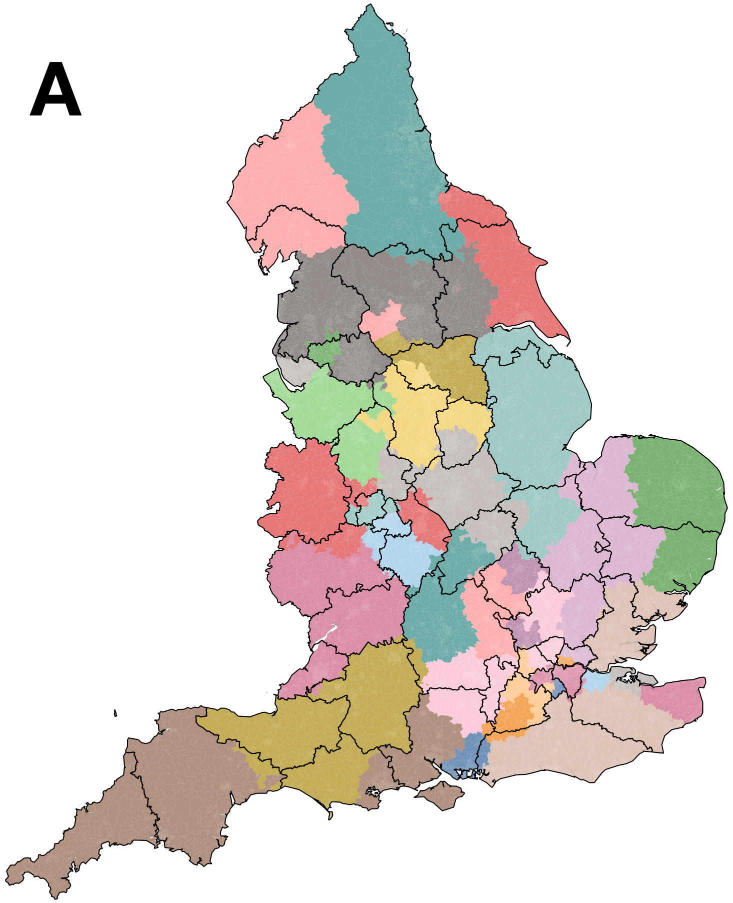
A
B
C

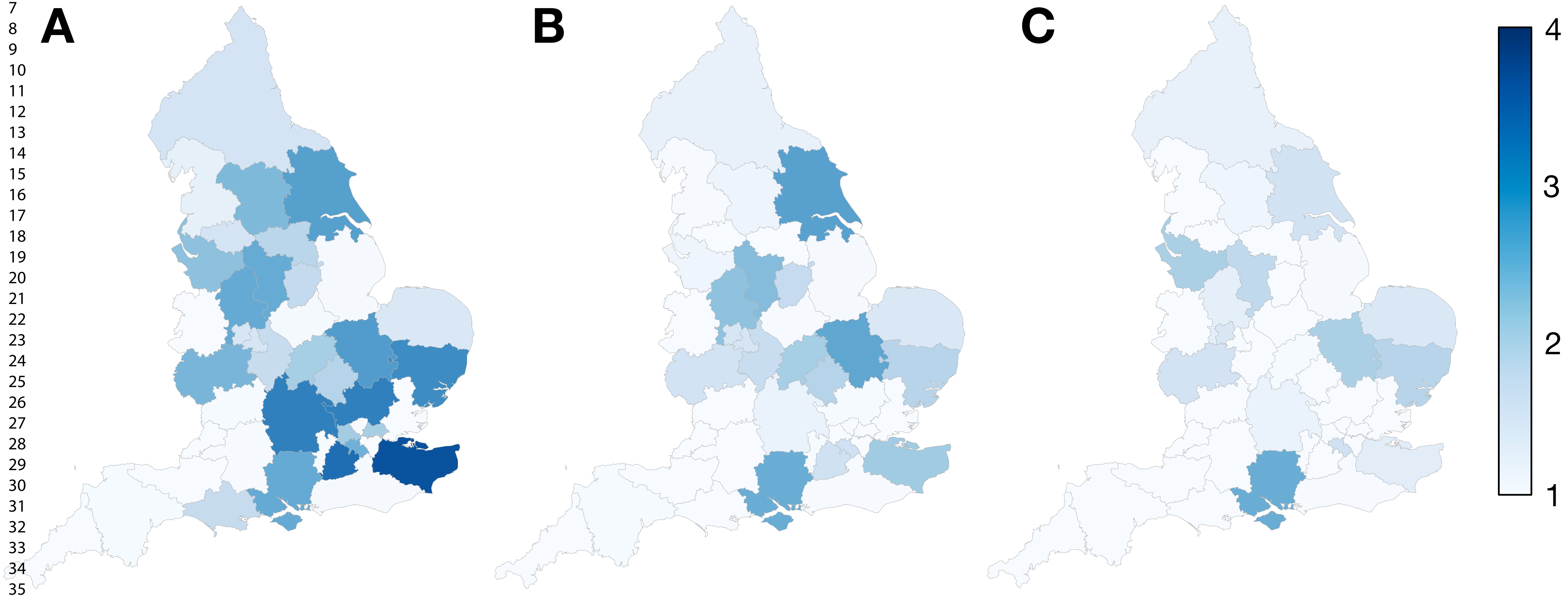
A
B
C
4
3
2
1

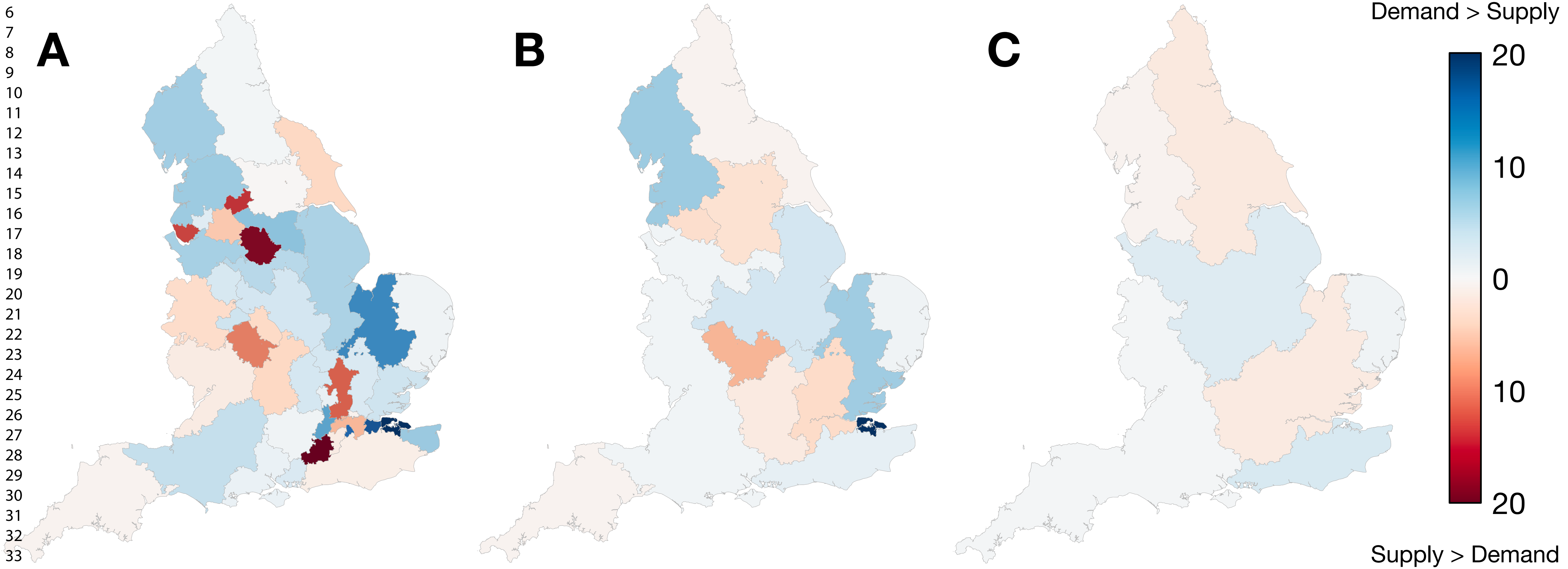
A
B
C
Demand > Supply
20
10
0
10
20
Supply > Demand

Appendix

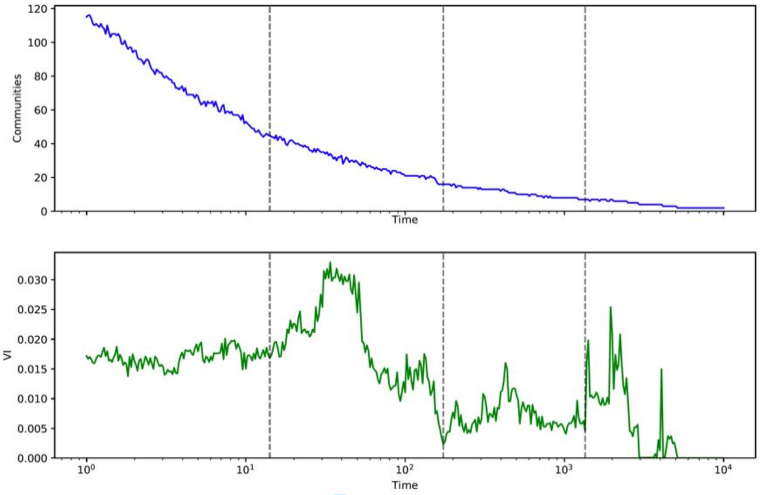

Figure 1b: Markov Multiscale Community Detection output showing the number of communities in the optimal network partition (top) and variation of information between partitions produced for Markov times from 1 to 10,000. Vertical lines indicate the three partitions of surgical communities selected for further review.

For peer review only

The RECORD statement – checklist of items, extended from the STROBE statement, that should be reported in observational studies using routinely collected health data.

	Item No.	STROBE items	Location in manuscript where items are reported	RECORD items	Location in manuscript where items are reported
**Title and abstract**					
	1	(a) Indicate the study's design with a commonly used term in the title or the abstract (b) Provide in the abstract an informative and balanced summary of what was done and what was found	Abstract and title (pages 1 and 3)	RECORD 1.1: The type of data used should be specified in the title or abstract. When possible, the name of the databases used should be included.<br><br>RECORD 1.2: If applicable, the geographic region and timeframe within which the study took place should be reported in the title or abstract.<br><br>RECORD 1.3: If linkage between databases was conducted for the study, this should be clearly stated in the title or abstract.	Abstract (page 3)<br><br>Abstract (page 3)<br><br>N/A
**Introduction**					
Background rationale	2	Explain the scientific background and rationale for the investigation being reported	Introduction (pages 5 and 6)		
Objectives	3	State specific objectives, including any prespecified hypotheses	Introduction (page 6)		
**Methods**					
Study Design	4	Present key elements of study design early in the paper	Methods (pages 6 and 7)		
Setting	5	Describe the setting, locations, and relevant dates, including periods of recruitment, exposure, follow-up, and data collection	Methods (pages 6 and 7)		

For peer review only

Participants	6	*(a) Cohort study* - Give the eligibility criteria, and the sources and methods of selection of participants. Describe methods of follow-up<br>*Case-control study* - Give the eligibility criteria, and the sources and methods of case ascertainment and control selection. Give the rationale for the choice of cases and controls<br>*Cross-sectional study* - Give the eligibility criteria, and the sources and methods of selection of participants<br><br>*(b) Cohort study* - For matched studies, give matching criteria and number of exposed and unexposed<br>*Case-control study* - For matched studies, give matching criteria and the number of controls per case	Methods (pages 6 and 7)	RECORD 6.1: The methods of study population selection (such as codes or algorithms used to identify subjects) should be listed in detail. If this is not possible, an explanation should be provided.<br><br>RECORD 6.2: Any validation studies of the codes or algorithms used to select the population should be referenced. If validation was conducted for this study and not published elsewhere, detailed methods and results should be provided.<br><br>RECORD 6.3: If the study involved linkage of databases, consider use of a flow diagram or other graphical display to demonstrate the data linkage process, including the number of individuals with linked data at each stage.	Methods (pages 6 and 7)<br><br>Methods (pages 6 and 7)<br><br>N/A
Variables	7	Clearly define all outcomes, exposures, predictors, potential confounders, and effect modifiers. Give diagnostic criteria, if applicable.	Methods (page 7)	RECORD 7.1: A complete list of codes and algorithms used to classify exposures, outcomes, confounders, and effect modifiers should be provided. If these cannot be reported, an explanation should be provided.	N/A
Data sources/ measurement	8	For each variable of interest, give sources of data and details of methods of assessment (measurement).<br>Describe comparability of assessment methods if there is more than one group	Methods (page 7)		

For peer review only

Bias	9	Describe any efforts to address potential sources of bias	Methods (pages 6, 7 and 8)		
Study size	10	Explain how the study size was arrived at	Methods (page 6)		
Quantitative variables	11	Explain how quantitative variables were handled in the analyses. If applicable, describe which groupings were chosen, and why	Methods (pages 7, 8 and 9)		
Statistical methods	12	(a) Describe all statistical methods, including those used to control for confounding<br>(b) Describe any methods used to examine subgroups and interactions<br>(c) Explain how missing data were addressed<br>(d) *Cohort study* - If applicable, explain how loss to follow-up was addressed<br>*Case-control study* - If applicable, explain how matching of cases and controls was addressed<br>*Cross-sectional study* - If applicable, describe analytical methods taking account of sampling strategy<br>(e) Describe any sensitivity analyses	Methods (pages 7, 8 and 9)		
Data access and cleaning methods		..		RECORD 12.1: Authors should describe the extent to which the investigators had access to the database population used to create the study population.	Methods (pages 6 and 7)

For peer review only

				RECORD 12.2: Authors should provide information on the data cleaning methods used in the study.	Methods (pages 6 and 7)
Linkage		..		RECORD 12.3: State whether the study included person-level, institutional-level, or other data linkage across two or more databases. The methods of linkage and methods of linkage quality evaluation should be provided.	N/A
**Results**					
Participants	13	(a) Report the numbers of individuals at each stage of the study (*e.g.*, numbers potentially eligible, examined for eligibility, confirmed eligible, included in the study, completing follow-up, and analysed)<br>(b) Give reasons for non-participation at each stage.<br>(c) Consider use of a flow diagram	Results (page 10)	RECORD 13.1: Describe in detail the selection of the persons included in the study (*i.e.,* study population selection) including filtering based on data quality, data availability and linkage. The selection of included persons can be described in the text and/or by means of the study flow diagram.	Methods (pages 6 and 7)
Descriptive data	14	(a) Give characteristics of study participants (*e.g.*, demographic, clinical, social) and information on exposures and potential confounders<br>(b) Indicate the number of participants with missing data for each variable of interest<br>(c) *Cohort study* - summarise follow-up time (*e.g.*, average and total amount)	Results (pages 10 and 11)		
Outcome data	15	*Cohort study* - Report numbers of outcome events or summary measures over time<br>*Case-control study* - Report numbers in each exposure			

For peer review only

		category, or summary measures of exposure<br>*Cross-sectional study* - Report numbers of outcome events or summary measures	Results (pages 10 and 11)		
Main results	16	(a) Give unadjusted estimates and, if applicable, confounder-adjusted estimates and their precision (e.g., 95% confidence interval). Make clear which confounders were adjusted for and why they were included<br>(b) Report category boundaries when continuous variables were categorized<br>(c) If relevant, consider translating estimates of relative risk into absolute risk for a meaningful time period	Results (pages 10 and 11)		
Other analyses	17	Report other analyses done—e.g., analyses of subgroups and interactions, and sensitivity analyses	Results (pages 10 and 11)		
**Discussion**					
Key results	18	Summarise key results with reference to study objectives	Discussion (page 12)		
Limitations	19	Discuss limitations of the study, taking into account sources of potential bias or imprecision. Discuss both direction and magnitude of any potential bias	Discussion (page 14)	RECORD 19.1: Discuss the implications of using data that were not created or collected to answer the specific research question(s). Include discussion of misclassification bias, unmeasured confounding, missing data, and changing eligibility over time, as they pertain to the study being reported.	Discussion (page 14)
Interpretation	20	Give a cautious overall interpretation of results considering objectives,	Discussion (page 14)		

For peer review only

		limitations, multiplicity of analyses, results from similar studies, and other relevant evidence			
Generalisability	21	Discuss the generalisability (external validity) of the study results	Discussion (pages 12, 13 and 14)		
**Other Information**					
Funding	22	Give the source of funding and the role of the funders for the present study and, if applicable, for the original study on which the present article is based	Page 16		
Accessibility of protocol, raw data, and programming code		..		RECORD 22.1: Authors should provide information on how to access any supplemental information such as the study protocol, raw data, or programming code.	Page 17

*Reference: Benchimol EI, Smeeth L, Guttmann A, Harron K, Moher D, Petersen I, Sørensen HT, von Elm E, Langan SM, the RECORD Working Committee. The REporting of studies Conducted using Observational Routinely-collected health Data (RECORD) Statement. *PLoS Medicine* 2015; in press.

*Checklist is protected under Creative Commons Attribution (CC BY) license.

For peer review only

BMJ Open

A New Geographic Model of Care to Manage the Post-COVID-19 Elective Surgery Aftershock in England: A Retrospective Observational Study

Journal:	*BMJ Open*
Manuscript ID	bmjopen-2020-042392.R1
Article Type:	Original research
Date Submitted by the Author:	29-Aug-2020

Complete List of Authors:	Clarke, Jonathan; Imperial College of Science Technology and Medicine, Department of Mathematics<br>Murray, Alice; Imperial College of Science Technology and Medicine, Department of Surgery and Cancer<br>Markar, Sheraz; Imperial College London, Department of Surgery and Cancer<br>Barahona, Mauricio; Imperial College of Science Technology and Medicine, Department of Mathematics<br>Kinross, James; Imperial College London, Division of Surgery

**Primary Subject Heading**:	Health policy
Secondary Subject Heading:	Surgery, Public health, Health services research
Keywords:	SURGERY, Health policy < HEALTH SERVICES ADMINISTRATION & MANAGEMENT, Organisation of health services < HEALTH SERVICES ADMINISTRATION & MANAGEMENT, PUBLIC HEALTH

SCHOLARONE™
Manuscripts

I, the Submitting Author has the right to grant and does grant on behalf of all authors of the Work (as defined in the below author licence), an exclusive licence and/or a non-exclusive licence for contributions from authors who are: i) UK Crown employees; ii) where BMJ has agreed a CC-BY licence shall apply, and/or iii) in accordance with the terms applicable for US Federal Government officers or employees acting as part of their official duties; on a worldwide, perpetual, irrevocable, royalty-free basis to BMJ Publishing Group Ltd ("BMJ") its licensees and where the relevant Journal is co-owned by BMJ to the co-owners of the Journal, to publish the Work in this journal and any other BMJ products and to exploit all rights, as set out in our licence.

The Submitting Author accepts and understands that any supply made under these terms is made by BMJ to the Submitting Author unless you are acting as an employee on behalf of your employer or a postgraduate student of an affiliated institution which is paying any applicable article publishing charge ("APC") for Open Access articles. Where the Submitting Author wishes to make the Work available on an Open Access basis (and intends to pay the relevant APC), the terms of reuse of such Open Access shall be governed by a Creative Commons licence – details of these licences and which Creative Commons licence will apply to this Work are set out in our licence referred to above.

Other than as permitted in any relevant BMJ Author's Self Archiving Policies, I confirm this Work has not been accepted for publication elsewhere, is not being considered for publication elsewhere and does not duplicate material already published. I confirm all authors consent to publication of this Work and authorise the granting of this licence.

For peer review only

A New Geographic Model of Care to Manage the Post-COVID-19 Elective Surgery Aftershock in England: A Retrospective Observational Study

Jonathan Clarke*, Alice Murray*, Sheraz Markar, Mauricio Barahona, James Kinross on behalf of the PanSurg Collaborative.

* These authors contributed equally to the study.

For peer review only

Jonathan M Clarke
Clinical Research Fellow, Department of Mathematics, Imperial College London, Huxley Building, South Kensington Campus, London, UK, SW7 2AZ
ORCID ID: 0000-0003-1495-7746

Alice Murray
General Surgical Registrar, Department of Surgery and Cancer, Imperial College London, London, UK, SW7 5NH
ORCID ID: 0000-0002-5058-0491

Sheraz Markar
NIHR Clinical Lecturer, Department of Surgery and Cancer, Imperial College London, London, UK, SW7 5NH

Mauricio Barahona
Professor of Mathematics, Department of Mathematics, Imperial College London, Huxley Building, South Kensington Campus, London, UK, SW7 2AZ
ORCID ID: 0000-0002-1089-5675

James Kinross
Senior Clinical Lecturer, Department of Surgery and Cancer, Imperial College London, London, UK, SW7 5NH

For peer review only - http://bmjopen.bmj.com/site/about/guidelines.xhtml

Correspondence to:

Jonathan Clarke

ORCID ID: 0000-0003-1495-7746

Department of Mathematics, Huxley Building, South Kensington Campus, London, SW7 2AZ, UK

j.clarke@imperial.ac.uk,

+44 (0) 7792 690037

Word Count: 4041

For peer review only

For peer review only - http://bmjopen.bmj.com/site/about/guidelines.xhtml

ABSTRACT

Objectives

The suspension of elective surgery during the COVID pandemic is unprecedented and has resulted in record volumes of patients waiting for operations. Novel approaches that maximise capacity and efficiency of surgical care are urgently required. This study applies Markov Multiscale Community Detection (MMCD), an unsupervised graph-based clustering framework, to identify new surgical care models based on pooled waiting lists delivered across an expanded network of surgical providers.

Design

Retrospective observational study using Hospital Episode Statistics.

Setting

Public and private hospitals providing surgical care to National Health Service (NHS) patients in England.

Participants

All adult patients resident in England undergoing NHS-funded planned surgical procedures between 1st April 2017 and 31st March 2018.

Main outcome measures

The identification of the most common planned surgical procedures in England (High Volume Procedures – HVP) and proportion of low, medium and high-risk patients undergoing each HVP. The mapping of hospitals providing surgical care onto optimised groupings based on patient usage data.

Results

A total of 7,811,891 planned operations were identified in 4,284,925 adults during the one-year period of our study. The 28 most common surgical procedures accounted for a combined 3,907,474 operations (50.0% of the total). 2,412,613 (61.7%) of these most

For peer review only - http://bmjopen.bmj.com/site/about/guidelines.xhtml

common procedures involved ‘low risk’ patients. Patients travelled an average of 11.3 km for these procedures. Based on the data, MMCD partitioned England into 45, 16 and 7 mutually exclusive and collectively exhaustive natural surgical communities of increasing coarseness. The coarser partitions into 16 and 7 surgical communities were shown to be associated with balanced supply and demand for surgical care within communities.

Conclusions

Pooled waiting lists for low risk elective procedures and patients across integrated, expanded natural surgical community networks have the potential to increase efficiency by innovatively flexing existing supply to better match demand.

Article Summary:

Strengths and Limitations of this Study

- The COVID-19 pandemic has significantly disrupted the provision of planned surgical care in hospitals across the world. Addressing the accumulated backlog of cases requires a new model of care whereby procedures are carried out at pace, while also responding to the dynamic risk of further COVID-19 outbreaks.
- This study utilises national, retrospective hospital administrative data relating to 7.8 million interventional procedures in 4.2 million adults.
- Markov Multiscale Community Detection, an unsupervised network clustering technique, is applied to understand how providers of surgical care may collaborate with one another based on prior patterns of surgical care delivery.
- The relative imbalances in supply and demand for surgical care within the identified surgical communities is quantified in order to determine the potential applicability of different scales of collaboration between care providers.
- While this study advances the potential role of collaboration between surgical centres to address the surgical backlog resulting from COVID-19, it does not address issues relating to local financial or logistical barriers to implementation of such a strategy.

For peer review only - http://bmjopen.bmj.com/site/about/guidelines.xhtml

For peer review only

For peer review only - http://bmjopen.bmj.com/site/about/guidelines.xhtml

Introduction

The COVID-19 pandemic put a global halt to the majority of elective surgery in order to manage the surge in patients requiring acute hospital services and ITU care.[1-4] It has been estimated that 28 million elective operations worldwide have been cancelled or postponed due to the pandemic.[5] Although the focus of public health organisations globally was rightly mounting an effective emergency response to the COVID-19 pandemic, the surgical ‘aftershock’ will therefore be unprecedented and yet to be fully appreciated. Millions of patients in the UK are already waiting for treatment and numbers increase daily as the diversion of resources continues.[6] Elective surgical services are gradually being re-introduced, aiming to treat waiting patients without risking the spread of COVID. Management strategies in the UK are currently focused on undertaking life-saving cancer operations in “clean” COVID-free hospitals or in hospital sites away from the acute care sites where COVID is more prevalent.[7 8] An immediate response to “catch up” and clear caseload will need to be undertaken, as well as adjusting to a “new normal”.

Waiting list numbers vary widely across the country and waiting times have increased in recent years[9]. To add complexity, there is also regional variation in the number of COVID infections and burden of COVID-related workload.[10,11] Therefore, in order to respond to the needs of a particular population, dynamic, flexible, regional solutions will be required to balance the reintroduction of services with careful COVID management.

Flexibility in the location where care is provided, according to patients’ clinical needs, has the potential to better match supply of services where there is appropriate demand. Patients can be treated more promptly if surgeons, hospitals and hospital delivery systems work together across provider networks, managing a centrally pooled workload. While some patients will need to be treated at specific locations (particularly high-risk patients or those requiring complex cancer care), there are other less complex procedures that could feasibly be performed by a range of qualified providers for patients who are able to travel.[12]

For peer review only - http://bmjopen.bmj.com/site/about/guidelines.xhtml

As the National Health Service (NHS) in England moves towards greater integration, there is an opportunity to break down arbitrary geographic boundaries and funding barriers, and bring together multiple providers of surgical care into 'surgical communities'. In such configurations, hospitals share a centrally managed waiting list for routine surgical procedures, and patients may receive surgery at any centre within the community of providers with the capacity to do so. There is a precedent for this approach, as a similar scheme was successfully piloted on a small scale in London.[13] Pooling available capacity between communities of surgical care providers may enable the efficient use of their collective available resources.

In this study we explore the potential of using flexible locations of care as a strategy to manage waiting lists. Firstly, we categorize the types of elective procedures and eligible patients into groups that would be amenable to undergoing surgery in any suitable location. Secondly, we identify from patient data existing community networks of surgical providers ('surgical communities') that collectively provide planned surgical care to similar geographic patient populations. Thirdly, we map these surgical communities against existing organizational configurations and model the effect on supply and demand when patients travel further for care.

Methods

All planned inpatient admissions to hospitals in England involving a surgical procedure were identified for adults resident in England from Hospital Episode Statistics from the 1st April 2017 to 31st March 2018. NHS-funded procedures conducted in non-NHS hospitals were included. For each admission, the first operative day was defined as the first day within an admission in which a surgical procedure was recorded. Procedures performed after the first operative day were excluded from the analysis. Where multiple procedures were performed on the first operative day, all of those procedures were counted to capture the fullest reliable representation of planned surgical activity. Inclusion of procedures after the first operative day is likely to include unplanned operations arising from surgical complications which are not identifiable as unplanned procedures in the data available.

For peer review only - http://bmjopen.bmj.com/site/about/guidelines.xhtml

All procedure codes describing diagnostic imaging, testing or rehabilitation (OPCS-4 codes beginning with U), the method of a procedure (Y) and site of a procedure (Z) were removed in addition to miscellaneous operations (X).[14] Procedures involving the concurrent extraction of a lens (C71) and insertion of a prosthetic lens (C75) were treated as a single procedure. Lower gastrointestinal diagnostic and therapeutic endoscopies frequently occurred concurrently or under codes with similar descriptions and were therefore grouped together. Conversely, diagnostic upper GI endoscopy (G45) was far more common than therapeutic endoscopies and was therefore treated separately.

Classification of Operative Risk

For each procedure, the age of the patient at the time of surgery was extracted. The modified Charlson comorbidity score of each patient was determined based on the presence of ICD-10 diagnosis codes extracted from their operative admission and all other recorded admissions to hospital for each patient in the 6 months prior to surgery.[15] Patients were then classified according to low, medium or high risk (for potential morbidity and mortality) by virtue of their age and Charlson Score (Table 1).

Identification of high-volume procedures

The total number of procedures performed for each 3-digit OPCS-4 code was calculated and sorted in descending order by volume. Those top procedures collectively accounting for more than 50% of the overall number of procedures were selected, and hereafter referred to as ‘High Volume Procedures’ (HVP).

Identification of hospital sites

The site in which a procedure was performed was identified from the SITETRET code of its associated admission. The postcodes of all sites in which procedures were performed were extracted from the site-level Estates Returns Information Collection.[16] Postcodes were converted to latitude and longitude coordinates. For all sites, the straight-line distance between all sites was calculated using the Haversine formula.[17] Where sites were within 1 km of one another, they were treated as a single merged site under the code and coordinates of the highest volume provider.

For peer review only - http://bmjopen.bmj.com/site/about/guidelines.xhtml

Calculation of Distance Travelled for Surgery

For each patient, the approximate location of their home was determined using the coordinates of the population-weighted centroid of their Lower Layer Super Output Area (LSOA) of residence.[18] LSOAs are mutually exclusive, collectively exhaustive geographic census divisions defined by the UK Office for National Statistics, of which there are 32,844 in England, with a mean population of 1,704 people, and is therefore similar in scale to Census Block Groups in the Unites States. The straight-line distance between the population-weighted centroid of the LSOA of residence of the patient and the site in which the procedure was performed was calculated according to the Haversine formula.

For each HVP, the total number of procedures performed was calculated. The number of patients classified as low, medium and high risk was calculated, along with the total number of sites undertaking the procedure and the average distance travelled for surgery. For each HVP, the total number of procedures performed by each site was calculated. To exclude providers who rarely perform a procedure, the highest volume providers who collectively accounted for 99% of procedures were identified and classified as providers of the HVP.

Identification of Surgical Communities

The proportion of patients presenting from each LSOA in England to each Regular Provider site for a HVP was calculated and a normalised cosine similarity matrix of LSOAs was computed (Equation 1).

$$similarity_{AB} = \frac{\sum_{i=1}^{n} \boldsymbol{A_i B_i}}{\sqrt{\sum_{i=1}^{n} \boldsymbol{A_i^2}}\sqrt{\sum_{i=1}^{n} \boldsymbol{B_i^2}}} \qquad (1)$$

Equation 1: Calculation of cosine similarity between LSOAs. A_i is the proportion of patients presenting to hospital site i resident in LSOA A; B_i is the proportion of patients presenting to hospital site i resident in LSOA B; and n is the total number of hospital sites in the dataset.

For peer review only - http://bmjopen.bmj.com/site/about/guidelines.xhtml

This matrix quantifies the similarity of patterns of presentation for HVPs between all LSOAs in England. It can be understood as the adjacency matrix of a dense, weighted network connecting LSOAs to one another according to the similarity in their patterns of presentation to hospital for HVPs.[19] This network was sparsened using the Relaxed Minimum Spanning Tree (RMST) technique, a method used elsewhere in applied network science to sparsen a dense, inhomogeneous network to preserve both local and global connectivity within a network. [20 21] This sparsened network was subsequently partitioned using Markov Multiscale Community Detection (MMCD) to produce partitions of the LSOAs according to shared patterns of presentation to hospital sites for HVPs.[22,23]

Description of Surgical Communities

The total number of procedures performed in each surgical community, and the total number of hospital sites was calculated. For each Sustainability and Transformation Partnership (STP - NHS organisational divisions of England into 44 regions responsible for developing local integration between primary and secondary care providers), the effective number of surgical communities active within its boundary was calculated using the Equivalent Market Size (the reciprocal of the Herfindahl Hirschman Index of market concentration) (Equation 2).[24]

$$EMS_i = 1/\sum_{j=1}^{N} s_{ij}^2 \qquad (2)$$

Equation 2: The Equivalent Market Size of STP_i. Here s_{ij} is the proportion of LSOAs in STP i contained within surgical community *j*, and *N* is the number of surgical communities in the partition.

Calculation of the Balance Between Supply and Demand Within Surgical Communities

Surgical communities were modelled as self-contained subdivisions of England containing LSOAs contributing cases requiring surgery (demand) and hospitals providing finite surgical capacity for those services (supply).[25] In this configuration, surgical

For peer review only - http://bmjopen.bmj.com/site/about/guidelines.xhtml

procedures for patients resident within a surgical community would be performed at a hospital site spatially located within the same surgical community. Within each surgical community, surgical demand was calculated as the total number of HVP cases performed for patients resident in LSOAs within the surgical community. Supply was calculated as the total number of HVP cases performed by sites located within the geographic boundary of the surgical community. The supply-demand mismatch was calculated as the percentage difference between supply and demand for each community. The median of the absolute value of the supply-demand mismatch was determined.

Patient and Public Involvement

We did not directly include PPI in this study, but the database used in the study was released following review by a panel including patient representatives.

Results

A total of 7,811,891 planned interventional procedures corresponding to 5,718,031 admissions involving 4,284,925 adult patients resident in England from 1st April 2017 to 31st March 2018 were identified. There procedures were performed at 530 NHS hospital sites and 162 different private provider sites. 1,210 different 3-digit OPCS codes were used.

28 types of procedure in Table 2 accounted for 3,907,474 operations, over half of all planned surgical procedures during the study period. These are denoted as High Volume Procedures (HVPs). Of these HVPs, 3,553,649 (90.9%) were performed in an NHS site, while 353,825 (9.9%) were performed in a non-NHS site. Collectively, diagnostic or therapeutic upper and lower gastrointestinal endoscopy accounted for 1.6 million procedures (20.3%). On average, procedures were performed on patients aged 61.4 years (SD = 16.7 years). 2,636,559 procedures were performed on patients with a Charlson comorbidity score of 0 (67.5%), while 997,765 procedures were performed on patients with a Charlson score of 1 or 2 (25.5%) and 273,150 procedures were performed on patients with a Charlson score of 3 or more (7.0%).

For peer review only - http://bmjopen.bmj.com/site/about/guidelines.xhtml

The mean distance travelled from a patient's residence to hospital for surgery was on average 11.3 km. Mean distances for the 28 HVPs ranged from 9.4 km for upper GI endoscopy to 16.2 km for spinal nerve root injection. 2,412,613 (61.7%) HVPs were performed in 'low risk' patients, 988,067 (25.3%) in 'medium risk' patients and 506,794 (13.0%) in 'high risk' patients. The proportion of procedures being performed on 'high risk' patients ranged from 1% for meniscal procedures to 52% for cystoscopy and resection of bladder lesions. In 22 out of 28 HVPs, more than 80% of patients were classified as 'low' or 'medium' risk.

Markov Multiscale Community Detection identified (see Figure 1b in the appendix) three robust community conformations of LSOAs consisting of 45 (Partition A), 16 (Partition B) and 7 (Partition C) surgical communities (Figure 1). Stable spatial motifs are observed across the three partitions.

Overlaid STP boundaries show variable agreement with surgical communities (Figure 1). Lower agreement is observed, for example in East Anglia, where surgical communities consistently partition in 'north-south' direction, while the STP boundary runs 'east to west'. Close agreement can be seen in Cornwall, where STPs are adjoining, based around surgical communities. The Hampshire and Isle of Wight STP, in the south of England, remains divided between more than three surgical communities in Partition C.

The median number of HVP cases performed in each community ranges from 78,998 in the finest partition (A) to 574,403 in the coarsest partition (C). In Partition A, the median number of surgical sites per community is 9, with an interquartile range from 9-17. In Partition B the median number of surgical sites per community in Partition B is 25, with an interquartile range of 19-44, while in Partition C, a median of 84 surgical sites are present per community, with an interquartile range of 56 to 98. In Partition A, STPs involved a median of 1.7 surgical communities, compared to 1.1 for Partition B and 1.0 for Partition C. Only the Hampshire and the Isle of Wight STP remains divided between more than three surgical communities in Partition C.

For peer review only - http://bmjopen.bmj.com/site/about/guidelines.xhtml

Supply and Demand Relationships within Surgical Communities

In Partition A, median absolute percentage difference between supply and demand for HVPs within surgical communities is 5.1%. 12 communities (27%) had absolute mismatches between supply and demand of more than 10%. These communities were located around conurbations in the North West of England and Greater London, with supply exceeding demand within cities, and demand exceeding supply in suburban communities. In Partition B, a supply demand mismatch exceeding 10% is only observed for the surgical community on the south of the Thames Estuary, where demand exceeds supply by 25%, indicating a role for nearby surgical sites in East London which lie outside of the community. In Partition C the percentage difference between supply and demand does not exceed 5% in any community.

Discussion

Hospital providers, policy makers and clinicians urgently require solutions for managing the COVID-19 elective surgical aftershock. This describes a state where COVID cases are in decline, in the context of strategically halted elective surgery and exponentially growing waiting lists. The extra-ordinary levels of demand for operations now requires radical new solutions to the way we organize and deliver surgical services. This study showed that there are existing hospital networks performing high volumes of low risk procedures for low risk, local patients. When we compare supply and demand for planned surgical care across England, the degree of mismatch varies widely, particularly around conurbations. Importantly, these data demonstrate that variation is reduced significantly when provider networks expand and smaller surgical communities coalesce into 16 larger geographic regions. We have identified a large group of potentially eligible, fit, lower risk patients who could be asked to travel greater distances than the existing median of 13 kms for their more minor surgery in order to shorten waiting times.

Central management of pooled waiting lists across an increased number of both NHS and non-NHS providers offers an opportunity for greater collaboration between surgical centres and a better distribution of workload. It would provide enhanced system resilience

For peer review only - http://bmjopen.bmj.com/site/about/guidelines.xhtml

in the context of future COVID outbreaks to continue planned surgery in dedicated clean sites.[8,26,27] The scheme may have additional benefits including increased patient choice, greater workforce flexibility and maximization of teams across areas, with increasing efficiency. There is a paucity of high quality data on the effects of pooled waiting lists.[28] Some evidence for their potential success has come from smaller, single site initiatives piloting internal pooling of cases distributed to consultants in the same department.[29] [30] [30] Surgical pooling has been used successfully in crises to achieve waiting-list targets with work done by non-consultant grade surgeons and cases shifted to the private sector. Surgical pooling has also been successful in matching existing supply to demand across transplant networks where donors are matched to recipients across larger regions, and sometimes between countries.[31] Greater choice and increased competition between providers for patients can be associated with reduced waiting times.[32] In this study we remain agnostic as to the means by which providers within a pooled list community should collaborate, and accept that the timing and mechanism of any collaboration should reflect the idiosyncrasies of local contexts over time – and is a determination which is best made by local providers.

The London Patient Choice Project (LPCP) was set up to reduce long waiting times for patients awaiting ophthalmic and minor general surgery procedures. Waiting lists were centrally pooled, managed and funded, with patients then given a choice on site of care in order to obtain earlier treatment. This lead to a convergence of waiting times across providers by relieving those hospitals with longer lists.[33] [13] Central purchasing of services was likely key to its success. On the strength of this pilot project, the English NHS undertook a national roll-out of patient choice, but without the central purchasing or coordination. 'Choose & Book' offered patients a choice of at least four hospitals which led some patients to attend a hospital other than the nearest one. Unpicking the effect of Choose and Book on waiting lists separately to other initiatives piloted at the time is complex, but it is likely that the setting of targets and strong performance management were key drivers on reducing waiting times rather than patient choice alone.[34]

For peer review only - http://bmjopen.bmj.com/site/about/guidelines.xhtml

In the UK patients generally favour the convenience and familiarity of a local provider. However, a MORI poll for the BMA showed that if faced with a long wait, 27% of people would travel anywhere in the United Kingdom for treatment by the NHS.[35] 78% of patients surveyed in the Isle of Wight were willing to travel to the mainland for elective surgery where the wait was shorter.[36] Greater patient travel has the potential to alleviate focal strain on services, but its practical application will require careful consideration. There are a number of barriers to travel – including patient mobility, age and risk as well as the cost of travel and the need for nearby family and friend support. In this study, selection of "low-risk patients and procedures" acts to mitigate some of these concerns, although the identification of operative risk based on procedure, age and Charlson score may be limited, and clearly in practice a patient-specific, case-by-case approach would be required. Government subsidization of travel would be an important intervention to reduce inequalities based on socio-economic status, education level, vulnerability or social exclusion.[35] However, in the London pilot there was no evidence of inequalities in uptake of the pooled list scheme by social class, educational attainment, income or ethnicity.[13] In the UK, with increasing centralization of complex care, particularly cancer care, patients are often already asked to travel further.[37] The applicability of a pooled-list surgical strategy varies according to the complexity of procedures and the need for in-person longitudinal follow-up with the operating centre. The finding that the majority of HVPs in this study are low complexity procedures, with limited need for onward follow-up supports the suitability of pooled provision for the HVPs identified.

In this study we identified a degree of variation in the extent to which demand for planned surgery within a community is met by the capacity of hospitals located in the same community. This is in addition to the current variation in waiting list lengths and COVID infection and hospitalisation rates. If variability could be reduced, or eliminated, then capacity planning is simplified.[38] This strategy fits with NHS England's broader integration strategy as outlined in the Five Year Forward view and continued in the expansion of STPs to become larger integrated delivery systems.

For peer review only - http://bmjopen.bmj.com/site/about/guidelines.xhtml

The extent to which demand for surgical care will change as a result of COVID-19 remains uncertain. General practitioners and patients may prefer strategies of watchful waiting for minor surgical conditions, consequently reducing demand. Similarly, periods of lower community COVID-19 transmission may result in increased referral for surgical services before another wave of the pandemic takes hold. Regional variation in standardised rates of planned surgical procedures indicate that reductions in surgical demand may perhaps be greater in areas of with lower pre-COVID-19 treatment thresholds and associated relative overuse.[39]

Similarly, the ability of hospitals to maintain pre-COVID-19 surgical capacity during and after the pandemic is uncertain. Recent research has demonstrated that some endoscopy departments in England maintained or even increased activity during the COVID-19 pandemic, while others stopped services entirely.[40] These findings indicate variation in local responses to the first wave COVID-19 and alludes to regional collaboration between surgical centres. In times of lower COVID-19 incidence, it could be expected that surgical supply may increase above pre-COVID-19 rates through provision of additional operating theatre capacity in evenings and weekends or the involvement of private sector care providers.[41] However, safely returning to baseline surgical capacity after a period of unprecedented disruption is a significant challenge in itself, and one where significant uncertainty remains. As a result of these uncertainties in future demand for, and supply of, surgical care, this study assumed future demand for planned surgical care would match historic demand from April 2017 to March 2018.

There are a number of limitations to the study. The COVID-19 epidemic is without precedent in recent history, so it was not possible to make substantially data-driven assumptions. The government have previously advised reducing national travel as a public health tool to limit COVID spread.[42] While our model does encourage patient mobility and could be criticized for the risk of further spread, it also facilitates more effective regional strategies to dedicate sites as COVID clean or dirty. Stringent infection control measures will be an essential part of any reintroduction of routine services. Currently there is mounting evidence that patients are not seeking out routine care due

For peer review only - http://bmjopen.bmj.com/site/about/guidelines.xhtml

For peer review only

to the perceived risk of COVID infection.[43] There is therefore a possibility that patients will choose not to undergo any elective procedures in the current climate, nor travel to an unknown hospital for that care. Pooled waiting lists are often disliked by surgeons who site the lack of autonomy and patient ownership with an increased risk of mis-diagnosis, unnecessary procedures listed, and unaddressed patient complexities.[44,45] These risks can, and should, be mitigated by ensuring clear standardised patient pathways, patient triage and suitability assessments, clarity in the named responsible surgeon and pathways for ongoing continuity of care. Virtual platforms have become increasingly available during COVID allowing remote consultation and triaging of patients prior to any procedures.[46] Finally, while we have identified a mismatch between current policy (STP boundaries) and practice (the natural networks of surgical providers), we appreciate that implementation of new integrated networks on a larger scale would require significant new resources and planning. A new system of funding flows, mechanisms for regional waiting list coordination and a cost per case mechanism or other financial incentive would be required to support this new model.

The NHS, despite being centrally funded, functions as a disparate collection of separate providers with their own priorities and resource constraints. In the COVID-19 pandemic, pre-existing structures of service delivery within the NHS were temporarily transformed. Primary care providers collaborated at a regional level to provide COVID care through a network of hubs while hospitals collaborated with one another to ensure some cancer care could continue at a smaller number of 'clean' hospital sites. As health systems across the world look to address an ever-growing backlog for planned care created by COVID-19, this trend of enhanced collaboration must continue. If the NHS is to overcome this backlog and cope with further waves of COVID, providers of surgical care must develop the means by which they may share a collective caseload for low-risk patients. What is certain is that the NHS, along with most other healthcare delivery systems, is having to make seismic changes to the way it works in order to best manage ongoing complexities. This study provides a solution with greater regional capacity flexibility with which to respond and adapt. Re-designing arbitrary geographical boundaries to follow expanded natural surgical community networks has the potential to increase efficiency by flexing

For peer review only - http://bmjopen.bmj.com/site/about/guidelines.xhtml

existing supply to meet demand. This, in addition to other key strategies, could have a profound effect on tackling the massive backlog of cases accruing during this deadly pandemic, thereby preventing further death, disability and reduced productivity from delayed surgery.

For peer review only

For peer review only - http://bmjopen.bmj.com/site/about/guidelines.xhtml

Author Contributions

JC and AM were involved in all aspects of the study. MB was involved in the development of the methodology and assisted in the formal analysis. SM, MB and JK were involved in the planning, conduct and reporting of the study. JC has had access to all the data in the study and all authors had final responsibility for the decision to submit for publication. JC attests that all listed authors meet authorship criteria and that no others meeting the criteria have been omitted.

Acknowledgements and Competing Interests

This article is independent research supported by grants from The Peter Sowerby Foundation and the National Institute for Health Research (NIHR) Imperial Patient Safety and Translational Research Centre (PSTRC) PSTRC_2016_004. Infrastructure support for this work was provided by the NIHR Imperial Biomedical Research Centre (BRC) 1215-20013. MB and JC acknowledge support from EPSRC grant EP/N014529/1 supporting the EPSRC Centre for Mathematics of Precision Healthcare. JC acknowledges support from the Wellcome Trust [215938/Z/19/Z]. Johnson and Johnson have supported the activities of the Pansurg collaborative with an educational grant. Data management was provided by the Big Data and Analytical Unit (BDAU) at the Institute of Global Health Innovation (IGHI), Imperial College London. The views expressed in this publication are those of the author(s) and not necessarily those of the NHS, the National Institute for Health Research or the Department of Health.

For peer review only

Dissemination declaration

Data as to hospital allocation to putative surgical communities will be made publicly available on www.healthdatascience.co.uk and will be disseminated to local care providers and commissioners.

Data Sharing Statement

Data used in this study were obtained from NHS Digital for the purpose of this work and may only be accessed through direct application to NHS Digital. Patient-level data is

For peer review only - http://bmjopen.bmj.com/site/about/guidelines.xhtml

required for the analyses conducted, and therefore sharing of data pertaining to this study is not possible. Data for surgical community assignments are available from the authors on request.

Transparency declaration

The lead author affirms that this manuscript is an honest, accurate, and transparent account of the study being reported; that no important aspects of the study have been omitted; and that any discrepancies from the study as planned have been explained.

Statement of Ethical Approval

This study received local ethical approval through the Imperial College Research Ethics Committee (17IC4178).

For peer review only

References

1. K. Turaga SG. Are we harming cancer patients by delaying their cancer surgery during the COVID-19 pandemic? *Annals of Surgery* 2020.
2. Mary E Brindle GD, Keith Lillemoe, Atul Gawande. Approaching surgical triage during the COVID-19 Pandemic. *Annals of Surgery* 2020.
3. American College of Surgeons. COVID-19: Elective Case Triage Guidelines for Surgical Care. https://www.facs.org/covid-19/clinical-guidance/elective-case. Published 2020, March 24.
4. Royal College of Surgeons. *COVID-19: Good Practice for Surgeons and Surgical Teams.* Royal College of Surgeons, England 2020.
5. Nepogodiev D, Bhangu A. Elective surgery cancellations due to the COVID-19 pandemic: global predictive modelling to inform surgical recovery plans. *The British journal of surgery.* 2020.
6. Brennan S. 'Mountain' waiting list will need five-year plan, says royal college. In. Vol 2020. Health Services Journal2020.
7. Brennan S. *Trusts told to create 'covid-free hubs' for cancer patients.* Health Services Journal 2020.
8. Kasivisvanathan V, Lindsay J, Rakhshani-moghadam S, et al. Evaluation of 30-day mortality for 500 patients undergoing non-emergency surgery in a COVID-19 cold site within a multicentre regional surgical network during the COVID-19 pandemic. *medRxiv.* 2020:2020.2006.2010.20115543.
9. National Audit Office. NHS waiting times for elective and cancer treatment. In: England DoHSCaN, ed2019.
10. NHS England. *NHS waiting times for elective and cancer treatment.* Department of Health & Social Care and NHS England;March 2019.
11. Challen R T-AK, Pitt M et al. Estimates of regional infectivity of COVID-19 in the United Kingdom following imposition of social distancing measures. *medRxiv.* 2020.
12. Dixon A RR, Appleby J, Burge P, Devlin N, Magee H. *Patient Choice. How Patients Choose and How Providers Respond.* . The King's Fund;2010.
13. Dawson D, Gravelle H, Jacobs R, Martin S, Smith PC. The effects of expanding patient choice of provider on waiting times: evidence from a policy experiment. *Health Econ.* 2007;16(2):113-128.
14. Health and Social Care Information Centre. *National Clinical Coding Standards OPCS-4. Accurate data for quality information* Health and Social Care Information Centre2019.
15. Quan H, Li B, Couris CM, et al. Updating and validating the Charlson comorbidity index and score for risk adjustment in hospital discharge abstracts using data from 6 countries. *Am J Epidemiol.* 2011;173(6):676-682.
16. NHS Digital. Estates Returns Information Collection. 2020.
17. Sinnott RW. Virtues of the Harversine *Sky and Telescope* 1984;68(2):158.
18. Office of National Statistics. Lower Layer Super Output Areas (December 2011) Population Weighted Centroids. https://geoportal.statistics.gov.uk/datasets/lower-

For peer review only - http://bmjopen.bmj.com/site/about/guidelines.xhtml

layer-super-output-areas-december-2011-population-weighted-centroids. Published 2019.
19. Clarke JM, Barahona M, Darzi AW. Defining Hospital Catchment Areas Using Multiscale Community Detection: A Case Study for Planned Orthopaedic Care in England. *bioRxiv.* 2019:619692.
20. Beguerisse-Díaz M, Vangelov B, Barahona M. Finding role communities in directed networks using Role-Based Similarity, Markov Stability and the Relaxed Minimum Spanning Tree. Paper presented at: 2013 IEEE Global Conference on Signal and Information Processing; 3-5 Dec. 2013.
21. Altuncu MT, Mayer E, Yaliraki SN, Barahona M. From free text to clusters of content in health records: an unsupervised graph partitioning approach. *Applied Network Science.* 2019;4(1):2.
22. Schaub MT, Delvenne J-C, Yaliraki SN, Barahona M. Markov dynamics as a zooming lens for multiscale community detection: non clique-like communities and the field-of-view limit. *PloS one.* 2012;7(2):e32210-e32210.
23. Lambiotte R, Delvenne J, Barahona M. Random Walks, Markov Processes and the Multiscale Modular Organization of Complex Networks. *IEEE Transactions on Network Science and Engineering.* 2014;1(2):76-90.
24. Rhoades SA. Market share inequality, the HHI, and other measures of the firm-composition of a market. *Review of Industrial Organization.* 1995;10(6):657-674.
25. Martin S, Rice N, Jacobs R, Smith P. The market for elective surgery: joint estimation of supply and demand. *J Health Econ.* 2007;26(2):263-285.
26. NHS England. Advice on maintaining cancer treatment during the COVID-19 response. In: Improvement NEaN, ed2020.
27. PanSurg. Exclusive: The three vital lessons Italian hospitals have learned in fighting covid-19. In: Health Services Journal, ed. Vol 2020. Health Services Journal
28. Ballini L, Negro A, Maltoni S, et al. Interventions to reduce waiting times for elective procedures. *Cochrane Database of Systematic Reviews.* 2015(2).
29. Leach P, Rutherford SA, King AT, Leggate JR. Generic waiting lists for routine spinal surgery. *Journal of the Royal Society of Medicine.* 2004;97(3):119-120.
30. John Appleby AH, Nancy Devlin. *What is the Real Cost of More Patient Choice?* : The King's Fund;2003.
31. Madsen M, Asmundsson P, Brekke IB, et al. Scandiatransplant: thirty years of cooperation in organ transplantation in the Nordic countries. *Clin Transpl.* 1998:121-131.
32. Siciliani L, Martin S. An empirical analysis of the impact of choice on waiting times. *Health Econ.* 2007;16(8):763-779.
33. Dawson D, Jacobs R, Martin S, Smith P. Is patient choice an effective mechanism to reduce waiting times? *Appl Health Econ Health Policy.* 2004;3(4):195-203.
34. Dixon A. *New Labour's Market Reforms - Patient Choice of Hospital.* 2011.
35. Damiani M, Propper C, Dixon J. Mapping choice in the NHS: cross sectional study of routinely collected data. *BMJ (Clinical research ed).* 2005;330(7486):284.

For peer review only

For peer review only - http://bmjopen.bmj.com/site/about/guidelines.xhtml

36. Ryan M, McIntosh E, Dean T, Old P. Trade-offs between location and waiting times in the provision of health care: the case of elective surgery on the Isle of Wight. *J Public Health Med.* 2000;22(2):202-210.
37. Aggarwal A, Lewis D, Mason M, Purushotham A, Sullivan R, van der Meulen J. Effect of patient choice and hospital competition on service configuration and technology adoption within cancer surgery: a national, population-based study. *Lancet Oncol.* 2017;18(11):1445-1453.
38. Pandit JJ, Pandit M, Reynard JM. Understanding waiting lists as the matching of surgical capacity to demand: are we wasting enough surgical time? *Anaesthesia.* 2010;65(6):625-640.
39. John Appleby VR, Francesca Frosini, Gwyn Bevan, Haiyan Gao, Tom Lyscom. Variations in Health Care. The King's Fund. https://www.kingsfund.org.uk/sites/default/files/field/field_publication_file/Variations-in-health-care-good-bad-inexplicable-report-The-Kings-Fund-April-2011.pdf. Published 2011. Accessed August, 2020.
40. Markar SR, Clarke J, Kinross J. Practice patterns of diagnostic upper gastrointestinal endoscopy during the initial COVID-19 outbreak in England. *Lancet Gastroenterol Hepatol.* 2020;5(9):804-805.
41. Permain N. COVID-19: Update on partnership working with the Independent Sector Providers and the Independent Healthcare Providers Network (IHPN) In: Teams RDRL, edAugust 2020.
42. The Health Protection (Coronavirus, Restrictions) (England) Regulations 2020.
43. Owens B. Reduced attendances and admissions: lessons from the covid crisis. In: Journal HS, ed. Vol 2020: HSJ; 2020.
44. Ramchandani M, Mirza S, Sharma A, Kirkby G. Pooled cataract waiting lists: views of hospital consultants, general practitioners and patients. *Journal of the Royal Society of Medicine.* 2002;95(12):598-600.
45. Position Statement on Pooled Waiting Lists [press release]. British Orthopaedic Association2013.
46. NHS England. Elective Care Transformation Programme 2020.

For peer review only - http://bmjopen.bmj.com/site/about/guidelines.xhtml

Figure captions:

Figure 1: Division of England into 45, 16 and 7 surgical communities (in colour). according to Markov Stability. STP boundaries are overlaid (black lines).

Figure 2: The equivalent number of surgical communities active in each STP as determined by the EMS. Areas of darker blue, 4 (e.g. East Anglia), represent those areas with greatest difference between surgical communities and STPs, whereas lighter blue shows greater agreement (e.g. Cornwall).

Figure 3: The absolute percentage difference between the number of patients undergoing surgery resident within a surgical community (demand) and the number of procedures performed by hospitals within the community (supply). Areas in blue represent those surgical communities where procedures performed on patients outnumber those performed by the local providers.

For peer review only

		Charlson Score		
		0	1-2	3+
**Age**	< 60	Low	Low	Medium
	60 - 74	Low	Medium	High
	75+	Medium	High	High

Table 1: Classification of low, medium and high-risk patients based on age and Charlson score.

For peer review only

For peer review only - http://bmjopen.bmj.com/site/about/guidelines.xhtml

		Patient risk			
**Procedure**	**Total no. of cases**	**Low risk (%)**	**Medium risk (%)**	**High risk (%)**	**Mean distance travelled, Kms**
Lower GI endoscopy	937,616	74.8	17.9	7.3	9.8
Upper GI endoscopy	650,133	66.9	22.1	10.9	9.4
Lens extraction + replacement	395,445	33.5	46.5	20.0	10.9
Excision of skin lesion	215,608	55.0	29.5	15.5	12.7
Injection/aspiration joint	142,562	71.6	20.9	7.5	12.6
Vitrectomy	132,938	39.9	44.1	16.1	13.2
Cystoscopy	130,114	56.4	26.2	17.4	11.8
Insertion central venous catheter	109,864	24.3	38.3	37.4	14.0
Coronary angiography	105,620	56.2	30.0	13.8	13.9
Dental extraction	101,435	91.6	5.8	2.5	11.5
Knee replacement	78,773	53.3	34.4	12.3	13.4
Bladder catheterisation or irrigation	71,552	42.5	32.7	24.8	12.8
Injection to bladder	67,167	34.3	29.8	35.9	11.5
Spinal facet joint injection	64,154	70.4	21.9	7.7	14.0
Cholecystectomy	61,790	80.5	13.8	5.7	11.8
Lymph node biopsy	60,674	34.8	34.4	30.8	14.9
Epidural or spinal injection	60,656	69.2	22.6	8.1	12.9
Inguinal hernia repair	58,943	72.6	19.9	7.5	10.9
Spinal nerve root injection	58,212	77.0	17.5	5.5	16.2
Knee meniscectomy/ meniscal repair	57,871	93.2	5.9	0.8	12.4
Hysteroscopy	52,360	90.9	6.4	2.7	9.9
Carpal tunnel release	48,245	70.7	22.2	7.1	11.0
Application/ removal of internal fixation of bone	46,771	84.6	11.9	3.5	15.7
Dental clearance	43,463	82.3	11.7	5.9	11.1
Partial breast excision	41,827	50.1	31.4	18.5	11.5
Bone marrow biopsy	38,369	39.8	34.8	25.5	15.6
Primary joint resurfacing	37,854	59.3	30.1	10.5	14.0
Cystoscopy + resection of bladder lesion	37,458	17.7	29.8	52.5	11.2

Table 2: The 28 procedures accounting for more than half of all elective surgical activity in England. The proportion of patients classified as low, medium and high risk according to Table 1 are shown, along with the mean distance travelled from a patient's LSOA of residence to the hospital site in which the procedure is performed.

For peer review only - http://bmjopen.bmj.com/site/about/guidelines.xhtml

Partition	A	B	C
Number of communities	45	16	7
Median number of cases per community	78998 (43628 - 118087)	214216 (122823 - 314022)	574403 (406465 - 679703)
Median number of treatment sites per community	9 (5 - 17)	25 (19 - 44)	84 (56 - 98)
Absolute supply:demand mismatch (%)	5.1 (2.9 - 10.2)	4.1 (1.0 - 5.7)	2.2 (1.0 - 2.9)

Table 3: Descriptive statistics for the three optimal partitions produced. Interquartile ranges are shown in parentheses where appropriate.

For peer review only

For peer review only - http://bmjopen.bmj.com/site/about/guidelines.xhtml

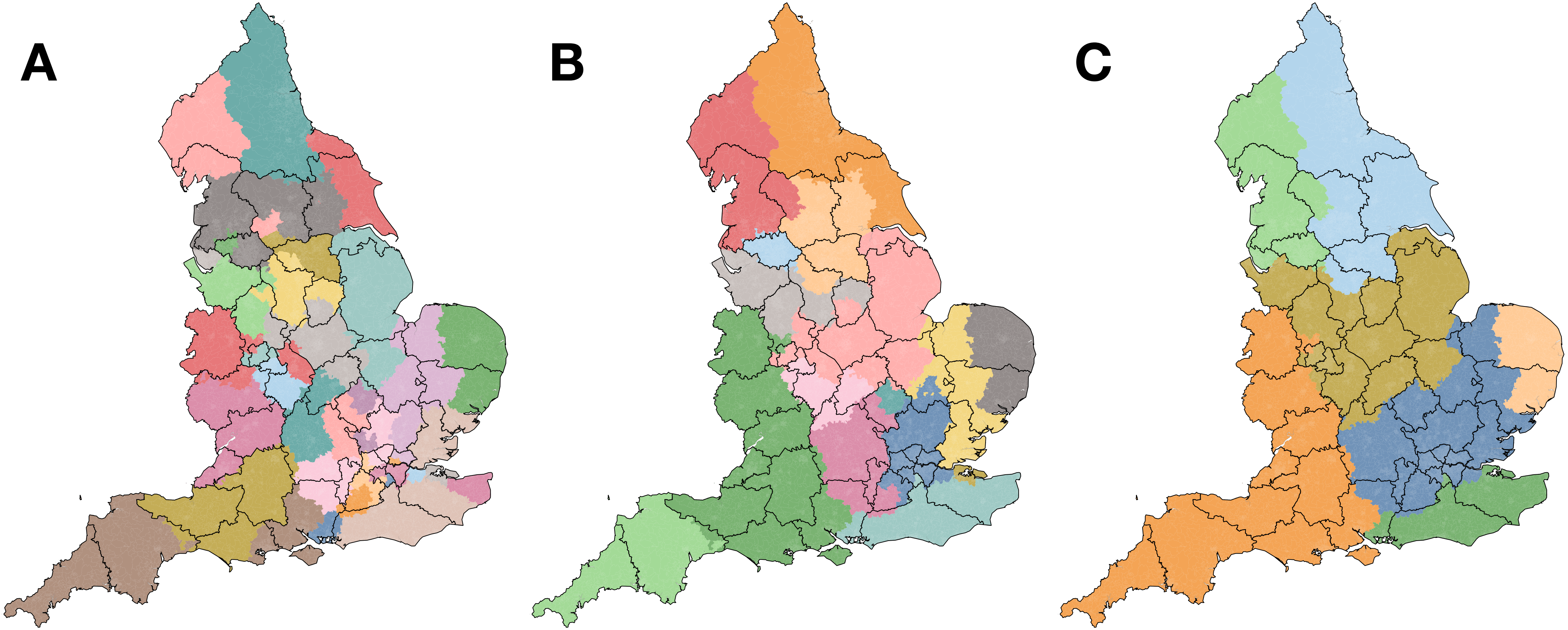
A
B
C

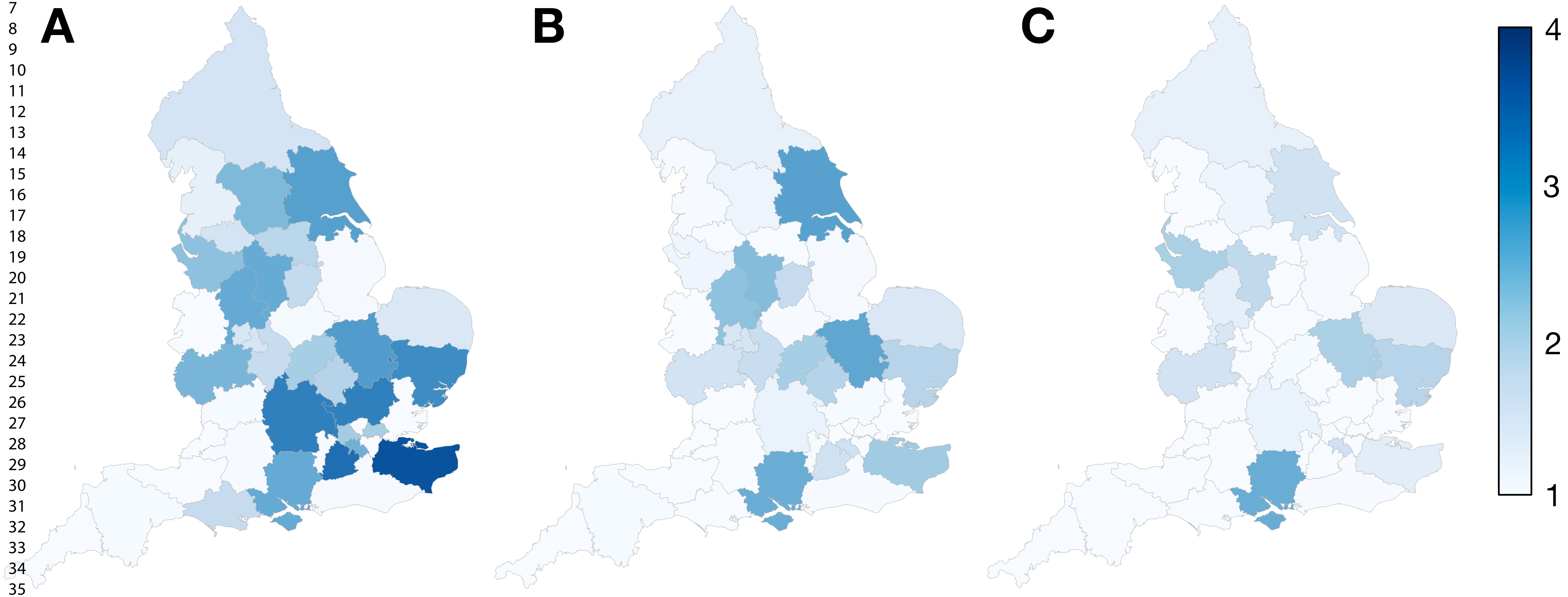
A
B
C
4
3
2
1

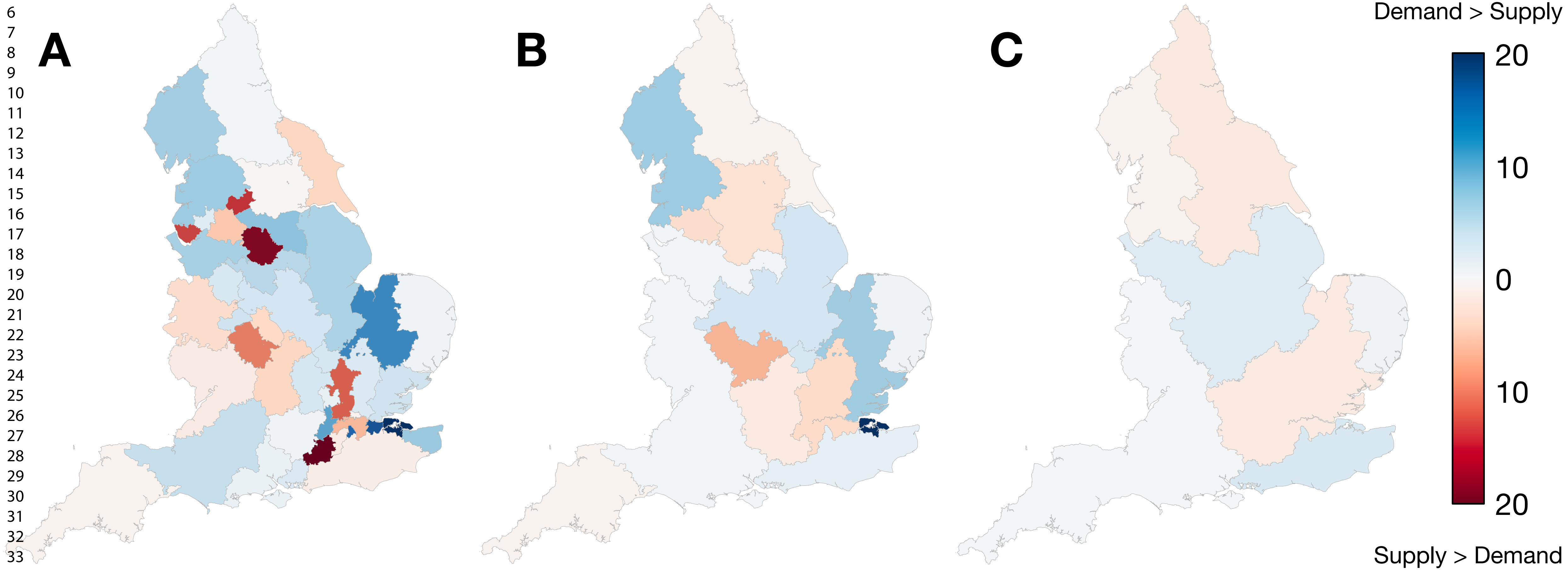
A
B
C
Demand > Supply
20
10
0
10
20
Supply > Demand

Appendix

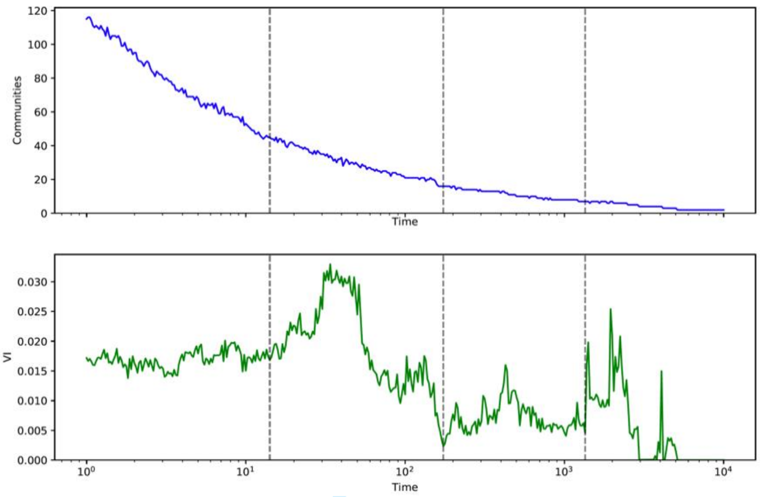

Supplemental Figure 1: Markov Multiscale Community Detection output showing the number of communities in the optimal network partition (top) and variation of information between partitions produced for Markov times from 1 to 10,000. Vertical lines indicate the three partitions of surgical communities selected for further review.

For peer review only

The RECORD statement – checklist of items, extended from the STROBE statement, that should be reported in observational studies using routinely collected health data.

	Item No.	STROBE items	Location in manuscript where items are reported	RECORD items	Location in manuscript where items are reported
**Title and abstract**					
	1	(a) Indicate the study's design with a commonly used term in the title or the abstract (b) Provide in the abstract an informative and balanced summary of what was done and what was found	Abstract and title (pages 1 and 3)	RECORD 1.1: The type of data used should be specified in the title or abstract. When possible, the name of the databases used should be included.<br><br>RECORD 1.2: If applicable, the geographic region and timeframe within which the study took place should be reported in the title or abstract.<br><br>RECORD 1.3: If linkage between databases was conducted for the study, this should be clearly stated in the title or abstract.	Abstract (page 3)<br><br>Abstract (page 3)<br><br>N/A
**Introduction**					
Background rationale	2	Explain the scientific background and rationale for the investigation being reported	Introduction (pages 5 and 6)		
Objectives	3	State specific objectives, including any prespecified hypotheses	Introduction (page 6)		
**Methods**					
Study Design	4	Present key elements of study design early in the paper	Methods (pages 6 and 7)		
Setting	5	Describe the setting, locations, and relevant dates, including periods of recruitment, exposure, follow-up, and data collection	Methods (pages 6 and 7)		

For peer review only

Participants	6	*(a) Cohort study* - Give the eligibility criteria, and the sources and methods of selection of participants. Describe methods of follow-up<br>*Case-control study* - Give the eligibility criteria, and the sources and methods of case ascertainment and control selection. Give the rationale for the choice of cases and controls<br>*Cross-sectional study* - Give the eligibility criteria, and the sources and methods of selection of participants<br><br>*(b) Cohort study* - For matched studies, give matching criteria and number of exposed and unexposed<br>*Case-control study* - For matched studies, give matching criteria and the number of controls per case	Methods (pages 6 and 7)	RECORD 6.1: The methods of study population selection (such as codes or algorithms used to identify subjects) should be listed in detail. If this is not possible, an explanation should be provided.<br><br>RECORD 6.2: Any validation studies of the codes or algorithms used to select the population should be referenced. If validation was conducted for this study and not published elsewhere, detailed methods and results should be provided.<br><br>RECORD 6.3: If the study involved linkage of databases, consider use of a flow diagram or other graphical display to demonstrate the data linkage process, including the number of individuals with linked data at each stage.	Methods (pages 6 and 7)<br><br>Methods (pages 6 and 7)<br><br>N/A
Variables	7	Clearly define all outcomes, exposures, predictors, potential confounders, and effect modifiers. Give diagnostic criteria, if applicable.	Methods (page 7)	RECORD 7.1: A complete list of codes and algorithms used to classify exposures, outcomes, confounders, and effect modifiers should be provided. If these cannot be reported, an explanation should be provided.	N/A
Data sources/ measurement	8	For each variable of interest, give sources of data and details of methods of assessment (measurement).<br>Describe comparability of assessment methods if there is more than one group	Methods (page 7)		

For peer review only

Bias	9	Describe any efforts to address potential sources of bias	Methods (pages 6, 7 and 8)		
Study size	10	Explain how the study size was arrived at	Methods (page 6)		
Quantitative variables	11	Explain how quantitative variables were handled in the analyses. If applicable, describe which groupings were chosen, and why	Methods (pages 7, 8 and 9)		
Statistical methods	12	(a) Describe all statistical methods, including those used to control for confounding<br>(b) Describe any methods used to examine subgroups and interactions<br>(c) Explain how missing data were addressed<br>(d) *Cohort study* - If applicable, explain how loss to follow-up was addressed<br>*Case-control study* - If applicable, explain how matching of cases and controls was addressed<br>*Cross-sectional study* - If applicable, describe analytical methods taking account of sampling strategy<br>(e) Describe any sensitivity analyses	Methods (pages 7, 8 and 9)		
Data access and cleaning methods		..		RECORD 12.1: Authors should describe the extent to which the investigators had access to the database population used to create the study population.	Methods (pages 6 and 7)

For peer review only

				RECORD 12.2: Authors should provide information on the data cleaning methods used in the study.	Methods (pages 6 and 7)
Linkage		..		RECORD 12.3: State whether the study included person-level, institutional-level, or other data linkage across two or more databases. The methods of linkage and methods of linkage quality evaluation should be provided.	N/A
**Results**					
Participants	13	(a) Report the numbers of individuals at each stage of the study (*e.g.*, numbers potentially eligible, examined for eligibility, confirmed eligible, included in the study, completing follow-up, and analysed)<br>(b) Give reasons for non-participation at each stage.<br>(c) Consider use of a flow diagram	Results (page 10)	RECORD 13.1: Describe in detail the selection of the persons included in the study (*i.e.,* study population selection) including filtering based on data quality, data availability and linkage. The selection of included persons can be described in the text and/or by means of the study flow diagram.	Methods (pages 6 and 7)
Descriptive data	14	(a) Give characteristics of study participants (*e.g.*, demographic, clinical, social) and information on exposures and potential confounders<br>(b) Indicate the number of participants with missing data for each variable of interest<br>(c) *Cohort study* - summarise follow-up time (*e.g.*, average and total amount)	Results (pages 10 and 11)		
Outcome data	15	*Cohort study* - Report numbers of outcome events or summary measures over time<br>*Case-control study* - Report numbers in each exposure			

For peer review only

		category, or summary measures of exposure<br>*Cross-sectional study* - Report numbers of outcome events or summary measures	Results (pages 10 and 11)		
Main results	16	(a) Give unadjusted estimates and, if applicable, confounder-adjusted estimates and their precision (e.g., 95% confidence interval). Make clear which confounders were adjusted for and why they were included<br>(b) Report category boundaries when continuous variables were categorized<br>(c) If relevant, consider translating estimates of relative risk into absolute risk for a meaningful time period	Results (pages 10 and 11)		
Other analyses	17	Report other analyses done—e.g., analyses of subgroups and interactions, and sensitivity analyses	Results (pages 10 and 11)		
**Discussion**					
Key results	18	Summarise key results with reference to study objectives	Discussion (page 12)		
Limitations	19	Discuss limitations of the study, taking into account sources of potential bias or imprecision. Discuss both direction and magnitude of any potential bias	Discussion (page 14)	RECORD 19.1: Discuss the implications of using data that were not created or collected to answer the specific research question(s). Include discussion of misclassification bias, unmeasured confounding, missing data, and changing eligibility over time, as they pertain to the study being reported.	Discussion (page 14)
Interpretation	20	Give a cautious overall interpretation of results considering objectives,	Discussion (page 14)		

For peer review only

		limitations, multiplicity of analyses, results from similar studies, and other relevant evidence			
Generalisability	21	Discuss the generalisability (external validity) of the study results	Discussion (pages 12, 13 and 14)		
**Other Information**					
Funding	22	Give the source of funding and the role of the funders for the present study and, if applicable, for the original study on which the present article is based	Page 16		
Accessibility of protocol, raw data, and programming code		..		RECORD 22.1: Authors should provide information on how to access any supplemental information such as the study protocol, raw data, or programming code.	Page 17

*Reference: Benchimol EI, Smeeth L, Guttmann A, Harron K, Moher D, Petersen I, Sørensen HT, von Elm E, Langan SM, the RECORD Working Committee. The REporting of studies Conducted using Observational Routinely-collected health Data (RECORD) Statement. *PLoS Medicine* 2015; in press.

*Checklist is protected under Creative Commons Attribution (CC BY) license.

For peer review only

BMJ Open

A New Geographic Model of Care to Manage the Post-COVID-19 Elective Surgery Aftershock in England: A Retrospective Observational Study

Journal:	*BMJ Open*
Manuscript ID	bmjopen-2020-042392.R2
Article Type:	Original research
Date Submitted by the Author:	29-Sep-2020

Complete List of Authors:	Clarke, Jonathan; Imperial College of Science Technology and Medicine, Department of Mathematics<br>Murray, Alice; Imperial College of Science Technology and Medicine, Department of Surgery and Cancer<br>Markar, Sheraz; Imperial College London, Department of Surgery and Cancer<br>Barahona, Mauricio; Imperial College of Science Technology and Medicine, Department of Mathematics<br>Kinross, James; Imperial College London, Division of Surgery

**Primary Subject Heading**:	Health policy
Secondary Subject Heading:	Surgery, Public health, Health services research
Keywords:	SURGERY, Health policy < HEALTH SERVICES ADMINISTRATION & MANAGEMENT, Organisation of health services < HEALTH SERVICES ADMINISTRATION & MANAGEMENT, PUBLIC HEALTH

SCHOLARONE™
Manuscripts

I, the Submitting Author has the right to grant and does grant on behalf of all authors of the Work (as defined in the below author licence), an exclusive licence and/or a non-exclusive licence for contributions from authors who are: i) UK Crown employees; ii) where BMJ has agreed a CC-BY licence shall apply, and/or iii) in accordance with the terms applicable for US Federal Government officers or employees acting as part of their official duties; on a worldwide, perpetual, irrevocable, royalty-free basis to BMJ Publishing Group Ltd ("BMJ") its licensees and where the relevant Journal is co-owned by BMJ to the co-owners of the Journal, to publish the Work in this journal and any other BMJ products and to exploit all rights, as set out in our licence.

The Submitting Author accepts and understands that any supply made under these terms is made by BMJ to the Submitting Author unless you are acting as an employee on behalf of your employer or a postgraduate student of an affiliated institution which is paying any applicable article publishing charge ("APC") for Open Access articles. Where the Submitting Author wishes to make the Work available on an Open Access basis (and intends to pay the relevant APC), the terms of reuse of such Open Access shall be governed by a Creative Commons licence – details of these licences and which Creative Commons licence will apply to this Work are set out in our licence referred to above.

Other than as permitted in any relevant BMJ Author's Self Archiving Policies, I confirm this Work has not been accepted for publication elsewhere, is not being considered for publication elsewhere and does not duplicate material already published. I confirm all authors consent to publication of this Work and authorise the granting of this licence.

For peer review only

A New Geographic Model of Care to Manage the Post-COVID-19 Elective Surgery Aftershock in England: A Retrospective Observational Study

Jonathan Clarke*, Alice Murray*, Sheraz Markar, Mauricio Barahona, James Kinross on behalf of the PanSurg Collaborative.

* These authors contributed equally to the study.

Jonathan M Clarke
Clinical Research Fellow, Department of Mathematics, Imperial College London, Huxley Building, South Kensington Campus, London, UK, SW7 2AZ
ORCID ID: 0000-0003-1495-7746

Alice Murray
General Surgical Registrar, Department of Surgery and Cancer, Imperial College London, London, UK, SW7 5NH
ORCID ID: 0000-0002-5058-0491

Sheraz Markar
NIHR Clinical Lecturer, Department of Surgery and Cancer, Imperial College London, London, UK, SW7 5NH

Mauricio Barahona
Professor of Mathematics, Department of Mathematics, Imperial College London, Huxley Building, South Kensington Campus, London, UK, SW7 2AZ
ORCID ID: 0000-0002-1089-5675

James Kinross
Senior Clinical Lecturer, Department of Surgery and Cancer, Imperial College London, London, UK, SW7 5NH

For peer review only - http://bmjopen.bmj.com/site/about/guidelines.xhtml

Correspondence to:

Jonathan Clarke

ORCID ID: 0000-0003-1495-7746

Department of Mathematics, Huxley Building, South Kensington Campus, London, SW7 2AZ, UK

j.clarke@imperial.ac.uk,

+44 (0) 7792 690037

Word Count: 4260

For peer review only

For peer review only - http://bmjopen.bmj.com/site/about/guidelines.xhtml

ABSTRACT

Objectives

The suspension of elective surgery during the COVID pandemic is unprecedented and has resulted in record volumes of patients waiting for operations. Novel approaches that maximise capacity and efficiency of surgical care are urgently required. This study applies Markov Multiscale Community Detection (MMCD), an unsupervised graph-based clustering framework, to identify new surgical care models based on pooled waiting lists delivered across an expanded network of surgical providers.

Design

Retrospective observational study using Hospital Episode Statistics.

Setting

Public and private hospitals providing surgical care to National Health Service (NHS) patients in England.

Participants

All adult patients resident in England undergoing NHS-funded planned surgical procedures between 1st April 2017 and 31st March 2018.

Main outcome measures

The identification of the most common planned surgical procedures in England (High Volume Procedures – HVP) and proportion of low, medium and high-risk patients undergoing each HVP. The mapping of hospitals providing surgical care onto optimised groupings based on patient usage data.

Results

A total of 7,811,891 planned operations were identified in 4,284,925 adults during the one-year period of our study. The 28 most common surgical procedures accounted for a combined 3,907,474 operations (50.0% of the total). 2,412,613 (61.7%) of these most

For peer review only - http://bmjopen.bmj.com/site/about/guidelines.xhtml

common procedures involved 'low risk' patients. Patients travelled an average of 11.3 km for these procedures. Based on the data, MMCD partitioned England into 45, 16 and 7 mutually exclusive and collectively exhaustive natural surgical communities of increasing coarseness. The coarser partitions into 16 and 7 surgical communities were shown to be associated with balanced supply and demand for surgical care within communities.

Conclusions

Pooled waiting lists for low risk elective procedures and patients across integrated, expanded natural surgical community networks have the potential to increase efficiency by innovatively flexing existing supply to better match demand.

Article Summary:

Strengths and Limitations of this Study

- The COVID-19 pandemic has significantly disrupted the provision of planned surgical care in hospitals across the world. Addressing the accumulated backlog of cases requires a new model of care whereby procedures are carried out at pace, while also responding to the dynamic risk of further COVID-19 outbreaks.
- This study utilises national, retrospective hospital administrative data relating to 7.8 million interventional procedures in 4.2 million adults.
- Markov Multiscale Community Detection, an unsupervised network clustering technique, is applied to understand how providers of surgical care may collaborate with one another based on prior patterns of surgical care delivery.
- The relative imbalances in supply and demand for surgical care within the identified surgical communities is quantified in order to determine the potential applicability of different scales of collaboration between care providers.
- While this study advances the potential role of collaboration between surgical centres to address the surgical backlog resulting from COVID-19, it does not address issues relating to local financial or logistical barriers to implementation of such a strategy.

For peer review only - http://bmjopen.bmj.com/site/about/guidelines.xhtml

For peer review only

For peer review only - http://bmjopen.bmj.com/site/about/guidelines.xhtml

Introduction

The COVID-19 pandemic put a global halt to the majority of elective surgery in order to manage the surge in patients requiring acute hospital services and ITU care.[1-4] It has been estimated that 28 million elective operations worldwide have been cancelled or postponed due to the pandemic.[5] Although the focus of public health organisations globally was rightly mounting an effective emergency response to the COVID-19 pandemic, the surgical 'aftershock' will therefore be unprecedented and yet to be fully appreciated. Millions of patients in the UK are already waiting for treatment and numbers increase daily as the diversion of resources continues.[6] Elective surgical services are gradually being re-introduced, aiming to treat waiting patients without risking the spread of COVID. Management strategies in the UK are currently focused on undertaking life-saving cancer operations in "clean" COVID-free hospitals or in hospital sites away from the acute care sites where COVID is more prevalent.[7 8] An immediate response to "catch up" and clear caseload will need to be undertaken, as well as adjusting to a "new normal".

Waiting list numbers vary widely across the country and waiting times have increased in recent years[9]. To add complexity, there is also regional variation in the number of COVID infections and burden of COVID-related workload.[10,11] Therefore, in order to respond to the needs of a particular population, dynamic, flexible, regional solutions will be required to balance the reintroduction of services with careful COVID management.

Flexibility in the location where care is provided, according to patients' clinical needs, has the potential to better match supply of services where there is appropriate demand. Patients can be treated more promptly if surgeons, hospitals and hospital delivery systems work together across provider networks, managing a centrally pooled workload. While some patients will need to be treated at specific locations (particularly high-risk patients or those requiring complex cancer care), there are other less complex procedures that could feasibly be performed by a range of qualified providers for patients who are able to travel.[12]

For peer review only - http://bmjopen.bmj.com/site/about/guidelines.xhtml

As the National Health Service (NHS) in England moves towards greater integration, there is an opportunity to break down arbitrary geographic boundaries and funding barriers, and bring together multiple providers of surgical care into ‘surgical communities’. In such configurations, hospitals share a centrally managed waiting list for routine surgical procedures, and patients may receive surgery at any centre within the community of providers with the capacity to do so. There is a precedent for this approach, as a similar scheme was successfully piloted on a small scale in London.[13] Pooling available capacity between communities of surgical care providers may enable the efficient use of their collective available resources.

In this study we explore the potential of using flexible locations of care as a strategy to manage waiting lists. Firstly, we categorize the types of elective procedures and eligible patients into groups that would be amenable to undergoing surgery in any suitable location. Secondly, we identify from patient data existing community networks of surgical providers (‘surgical communities’) that collectively provide planned surgical care to similar geographic patient populations. Thirdly, we map these surgical communities against existing organizational configurations and model the effect on supply and demand when patients travel further for care.

Methods

All planned inpatient admissions to hospitals in England involving a surgical procedure were identified for adults resident in England from Hospital Episode Statistics from the 1st April 2017 to 31st March 2018. NHS-funded procedures conducted in non-NHS hospitals were included. For each admission, the first operative day was defined as the first day within an admission in which a surgical procedure was recorded. Procedures performed after the first operative day were excluded from the analysis. Where multiple procedures were performed on the first operative day, all of those procedures were counted to capture the fullest reliable representation of planned surgical activity. Inclusion of procedures after the first operative day is likely to include unplanned operations arising from surgical complications which are not identifiable as unplanned procedures in the data available.

For peer review only - http://bmjopen.bmj.com/site/about/guidelines.xhtml

All procedure codes describing diagnostic imaging, testing or rehabilitation (OPCS-4 codes beginning with U), the method of a procedure (Y) and site of a procedure (Z) were removed in addition to miscellaneous operations (X).[14] Procedures involving the concurrent extraction of a lens (C71) and insertion of a prosthetic lens (C75) were treated as a single procedure. Lower gastrointestinal diagnostic and therapeutic endoscopies frequently occurred concurrently or under codes with similar descriptions and were therefore grouped together. Conversely, diagnostic upper GI endoscopy (G45) was far more common than therapeutic endoscopies and was therefore treated separately.

Classification of Operative Risk

For each procedure, the age of the patient at the time of surgery was extracted. The modified Charlson comorbidity score of each patient was determined based on the presence of ICD-10 diagnosis codes extracted from their operative admission and all other recorded admissions to hospital for each patient in the 6 months prior to surgery.[15] Patients were then classified according to low, medium or high risk (for potential morbidity and mortality) by virtue of their age and Charlson Score (Table 1).

Identification of high-volume procedures

The total number of procedures performed for each 3-digit OPCS-4 code was calculated and sorted in descending order by volume. Those top procedures collectively accounting for more than 50% of the overall number of procedures were selected, and hereafter referred to as 'High Volume Procedures' (HVP).

Identification of hospital sites

The site in which a procedure was performed was identified from the SITETRET code of its associated admission. The postcodes of all sites in which procedures were performed were extracted from the site-level Estates Returns Information Collection.[16] Postcodes were converted to latitude and longitude coordinates. For all sites, the straight-line distance between all sites was calculated using the Haversine formula.[17] Where sites were within 1 km of one another, they were treated as a single merged site under the code and coordinates of the highest volume provider.

For peer review only - http://bmjopen.bmj.com/site/about/guidelines.xhtml

Calculation of Distance Travelled for Surgery

For each patient, the approximate location of their home was determined using the coordinates of the population-weighted centroid of their Lower Layer Super Output Area (LSOA) of residence.[18] LSOAs are mutually exclusive, collectively exhaustive geographic census divisions defined by the UK Office for National Statistics, of which there are 32,844 in England, with a mean population of 1,704 people, and is therefore similar in scale to Census Block Groups in the Unites States. The straight-line distance between the population-weighted centroid of the LSOA of residence of the patient and the site in which the procedure was performed was calculated according to the Haversine formula.

For each HVP, the total number of procedures performed was calculated. The number of patients classified as low, medium and high risk was calculated, along with the total number of sites undertaking the procedure and the average distance travelled for surgery. For each HVP, the total number of procedures performed by each site was calculated. To exclude providers who rarely perform a procedure, the highest volume providers who collectively accounted for 99% of procedures were identified and classified as providers of the HVP.

Identification of Surgical Communities

The proportion of patients presenting from each LSOA in England to each Regular Provider site for a HVP was calculated and a normalised cosine similarity matrix of LSOAs was computed (Equation 1).

$$similarity_{AB} = \frac{\sum_{i=1}^{n} \boldsymbol{A_i B_i}}{\sqrt{\sum_{i=1}^{n} \boldsymbol{A_i^2}}\sqrt{\sum_{i=1}^{n} \boldsymbol{B_i^2}}} \qquad (1)$$

Equation 1: Calculation of cosine similarity between LSOAs. A_i is the proportion of patients presenting to hospital site i resident in LSOA A; B_i is the proportion of patients presenting to hospital site i resident in LSOA B; and n is the total number of hospital sites in the dataset.

For peer review only - http://bmjopen.bmj.com/site/about/guidelines.xhtml

This matrix quantifies the similarity of patterns of presentation for HVPs between all LSOAs in England. It can be understood as the adjacency matrix of a dense, weighted network connecting LSOAs to one another according to the similarity in their patterns of presentation to hospital for HVPs.[19] This network was sparsened using the Relaxed Minimum Spanning Tree (RMST) technique, a method used elsewhere in applied network science to sparsen a dense, inhomogeneous network to preserve both local and global connectivity within a network. [20 21] This sparsened network was subsequently partitioned using Markov Multiscale Community Detection (MMCD) to produce partitions of the LSOAs according to shared patterns of presentation to hospital sites for HVPs.[22,23]

Description of Surgical Communities

The total number of procedures performed in each surgical community, and the total number of hospital sites was calculated. For each Sustainability and Transformation Partnership (STP - NHS organisational divisions of England into 44 regions responsible for developing local integration between primary and secondary care providers), the effective number of surgical communities active within its boundary was calculated using the Equivalent Market Size (the reciprocal of the Herfindahl Hirschman Index of market concentration) (Equation 2).[24]

$$EMS_i = 1/\sum_{j=1}^{N} s_{ij}^2 \qquad (2)$$

Equation 2: The Equivalent Market Size of STP_i. Here s_{ij} is the proportion of LSOAs in STP i contained within surgical community *j*, and *N* is the number of surgical communities in the partition.

Calculation of the Balance Between Supply and Demand Within Surgical Communities

Surgical communities were modelled as self-contained subdivisions of England containing LSOAs contributing cases requiring surgery (demand) and hospitals providing finite surgical capacity for those services (supply).[25] In this configuration, surgical

For peer review only - http://bmjopen.bmj.com/site/about/guidelines.xhtml

procedures for patients resident within a surgical community would be performed at a hospital site spatially located within the same surgical community. Within each surgical community, surgical demand was calculated as the total number of HVP cases performed for patients resident in LSOAs within the surgical community. Supply was calculated as the total number of HVP cases performed by sites located within the geographic boundary of the surgical community. The supply-demand mismatch was calculated as the percentage difference between supply and demand for each community. The median of the absolute value of the supply-demand mismatch was determined.

Patient and Public Involvement

We did not directly include PPI in this study, but the database used in the study was released following review by a panel including patient representatives.

Results

A total of 7,811,891 planned interventional procedures corresponding to 5,718,031 admissions involving 4,284,925 adult patients resident in England from 1st April 2017 to 31st March 2018 were identified. There procedures were performed at 530 NHS hospital sites and 162 different private provider sites. 1,210 different 3-digit OPCS codes were used.

28 types of procedure in Table 2 accounted for 3,907,474 operations, over half of all planned surgical procedures during the study period. These are denoted as High Volume Procedures (HVPs). Of these HVPs, 3,553,649 (90.9%) were performed in an NHS site, while 353,825 (9.9%) were performed in a non-NHS site. Collectively, diagnostic or therapeutic upper and lower gastrointestinal endoscopy accounted for 1.6 million procedures (20.3%). On average, procedures were performed on patients aged 61.4 years (SD = 16.7 years). 2,636,559 procedures were performed on patients with a Charlson comorbidity score of 0 (67.5%), while 997,765 procedures were performed on patients with a Charlson score of 1 or 2 (25.5%) and 273,150 procedures were performed on patients with a Charlson score of 3 or more (7.0%).

For peer review only - http://bmjopen.bmj.com/site/about/guidelines.xhtml

The mean distance travelled from a patient's residence to hospital for surgery was on average 11.3 km. Mean distances for the 28 HVPs ranged from 9.4 km for upper GI endoscopy to 16.2 km for spinal nerve root injection. 2,412,613 (61.7%) HVPs were performed in 'low risk' patients, 988,067 (25.3%) in 'medium risk' patients and 506,794 (13.0%) in 'high risk' patients. The proportion of procedures being performed on 'high risk' patients ranged from 1% for meniscal procedures to 52% for cystoscopy and resection of bladder lesions. In 22 out of 28 HVPs, more than 80% of patients were classified as 'low' or 'medium' risk.

Markov Multiscale Community Detection identified (see Supplementary Figure 1 in the appendix) three robust community conformations of LSOAs consisting of 45 (Partition A), 16 (Partition B) and 7 (Partition C) surgical communities (Figure 1). Stable spatial motifs are observed across the three partitions.

Overlaid STP boundaries show variable agreement with surgical communities (Figure 1). Lower agreement is observed, for example in East Anglia, where surgical communities consistently partition in 'north-south' direction, while the STP boundary runs 'east to west'. Close agreement can be seen in Cornwall, where STPs are adjoining, based around surgical communities. The Hampshire and Isle of Wight STP, in the south of England, remains divided between more than three surgical communities in Partition C.

The median number of HVP cases performed in each community ranges from 78,998 in the finest partition (A) to 574,403 in the coarsest partition (C) (Table 3). In Partition A, the median number of surgical sites per community is 9, with an interquartile range from 9-17. In Partition B the median number of surgical sites per community in Partition B is 25, with an interquartile range of 19-44, while in Partition C, a median of 84 surgical sites are present per community, with an interquartile range of 56 to 98. In Partition A, STPs involved a median of 1.7 surgical communities, compared to 1.1 for Partition B and 1.0 for Partition C. Only the Hampshire and the Isle of Wight STP remains divided between more than three surgical communities in Partition C (Figure 2).

For peer review only - http://bmjopen.bmj.com/site/about/guidelines.xhtml

Supply and Demand Relationships within Surgical Communities

In Partition A, median absolute percentage difference between supply and demand for HVPs within surgical communities is 5.1%. 12 communities (27%) had absolute mismatches between supply and demand of more than 10% (Table 3). These communities were located around conurbations in the North West of England and Greater London, with supply exceeding demand within cities, and demand exceeding supply in suburban communities (Figure 3). In Partition B, a supply demand mismatch exceeding 10% is only observed for the surgical community on the south of the Thames Estuary, where demand exceeds supply by 25%, indicating a role for nearby surgical sites in East London which lie outside of the community. In Partition C the percentage difference between supply and demand does not exceed 5% in any community.

Discussion

Hospital providers, policy makers and clinicians urgently require solutions for managing the COVID-19 elective surgical aftershock. This describes a state where COVID cases are in decline, in the context of strategically halted elective surgery and exponentially growing waiting lists. The extra-ordinary levels of demand for operations now requires radical new solutions to the way we organize and deliver surgical services. This study showed that there are existing hospital networks performing high volumes of low risk procedures for low risk, local patients. When we compare supply and demand for planned surgical care across England, the degree of mismatch varies widely, particularly around conurbations. Importantly, these data demonstrate that variation is reduced significantly when provider networks expand and smaller surgical communities coalesce into 16 larger geographic regions. We have identified a large group of potentially eligible, fit, lower risk patients who could be asked to travel greater distances than the existing median of 13 kms for their more minor surgery in order to shorten waiting times.

Central management of pooled waiting lists across an increased number of both NHS and non-NHS providers offers an opportunity for greater collaboration between surgical centres and a better distribution of workload. It would provide enhanced system resilience

For peer review only - http://bmjopen.bmj.com/site/about/guidelines.xhtml

in the context of future COVID outbreaks to continue planned surgery in dedicated clean sites.[8,26,27] The scheme may have additional benefits including increased patient choice, greater workforce flexibility and maximization of teams across areas, with increasing efficiency. There is a paucity of high quality data on the effects of pooled waiting lists.[28] Some evidence for their potential success has come from smaller, single site initiatives piloting internal pooling of cases distributed to consultants in the same department.[29] [30] [30] Surgical pooling has been used successfully in crises to achieve waiting-list targets with work done by non-consultant grade surgeons and cases shifted to the private sector. Surgical pooling has also been successful in matching existing supply to demand across transplant networks where donors are matched to recipients across larger regions, and sometimes between countries.[31] Greater choice and increased competition between providers for patients can be associated with reduced waiting times.[32] In this study we remain agnostic as to the means by which providers within a pooled list community should collaborate, and accept that the timing and mechanism of any collaboration should reflect the idiosyncrasies of local contexts over time – and is a determination which is best made by local providers.

The London Patient Choice Project (LPCP) was set up to reduce long waiting times for patients awaiting ophthalmic and minor general surgery procedures. Waiting lists were centrally pooled, managed and funded, with patients then given a choice on site of care in order to obtain earlier treatment. This lead to a convergence of waiting times across providers by relieving those hospitals with longer lists.[33,13] Central purchasing of services was likely key to its success. On the strength of this pilot project, the English NHS undertook a national roll-out of patient choice, but without the central purchasing or coordination. 'Choose & Book' offered patients a choice of at least four hospitals which led some patients to attend a hospital other than the nearest one. Unpicking the effect of Choose and Book on waiting lists separately to other initiatives piloted at the time is complex, but it is likely that the setting of targets and strong performance management were key drivers on reducing waiting times rather than patient choice alone.[34]

For peer review only - http://bmjopen.bmj.com/site/about/guidelines.xhtml

In the UK patients generally favour the convenience and familiarity of a local provider. However, a MORI poll for the BMA showed that if faced with a long wait, 27% of people would travel anywhere in the United Kingdom for treatment by the NHS.[35] 78% of patients surveyed in the Isle of Wight were willing to travel to the mainland for elective surgery where the wait was shorter.[36] Greater patient travel has the potential to alleviate focal strain on services, but its practical application will require careful consideration. There are a number of barriers to travel – including patient mobility, age and risk as well as the cost of travel and the need for nearby family and friend support. In this study, selection of "low-risk patients and procedures" acts to mitigate some of these concerns, although the identification of operative risk based on procedure, age and Charlson score may be limited, and clearly in practice a patient-specific, case-by-case approach would be required. Government subsidization of travel would be an important intervention to reduce inequalities based on socio-economic status, education level, vulnerability or social exclusion.[35] However, in the London pilot there was no evidence of inequalities in uptake of the pooled list scheme by social class, educational attainment, income or ethnicity.[13] In the UK, with increasing centralization of complex care, particularly cancer care, patients are often already asked to travel further.[37] The applicability of a pooled-list surgical strategy varies according to the complexity of procedures and the need for in-person longitudinal follow-up with the operating centre. The finding that the majority of HVPs in this study are low complexity procedures, with limited need for onward follow-up supports the suitability of pooled provision for the HVPs identified.

In this study we identified a degree of variation in the extent to which demand for planned surgery within a community is met by the capacity of hospitals located in the same community. This is in addition to the current variation in waiting list lengths and COVID infection and hospitalisation rates. If variability could be reduced, or eliminated, then capacity planning is simplified.[38] This strategy fits with NHS England's broader integration strategy as outlined in the Five Year Forward view and continued in the expansion of STPs to become larger integrated delivery systems.

For peer review only - http://bmjopen.bmj.com/site/about/guidelines.xhtml

The extent to which demand for surgical care will change as a result of COVID-19 remains uncertain. General practitioners and patients may prefer strategies of watchful waiting for minor surgical conditions, consequently reducing demand. Similarly, periods of lower community COVID-19 transmission may result in increased referral for surgical services before another wave of the pandemic takes hold. Regional variation in standardised rates of planned surgical procedures indicate that reductions in surgical demand may perhaps be greater in areas of with lower pre-COVID-19 treatment thresholds and associated relative overuse.[39]

Similarly, the ability of hospitals to maintain pre-COVID-19 surgical capacity during and after the pandemic is uncertain. Recent research has demonstrated that some endoscopy departments in England maintained or even increased activity during the COVID-19 pandemic, while others stopped services entirely.[40] These findings indicate variation in local responses to the first wave COVID-19 and alludes to regional collaboration between surgical centres. In times of lower COVID-19 incidence, it could be expected that surgical supply may increase above pre-COVID-19 rates through provision of additional operating theatre capacity in evenings and weekends or the involvement of private sector care providers.[41] However, safely returning to baseline surgical capacity after a period of unprecedented disruption is a significant challenge in itself, and one where significant uncertainty remains. As a result of these uncertainties in future demand for, and supply of, surgical care, this study assumed future demand for planned surgical care would match historic demand from April 2017 to March 2018.

There are a number of limitations to the study. The COVID-19 epidemic is without precedent in recent history, so it was not possible to make substantially data-driven assumptions. The government have previously advised reducing national travel as a public health tool to limit COVID spread.[42] While our model does encourage patient mobility and could be criticized for the risk of further spread, it also facilitates more effective regional strategies to dedicate sites as COVID clean or dirty. Stringent infection control measures will be an essential part of any reintroduction of routine services. Currently there is mounting evidence that patients are not seeking out routine care due

For peer review only - http://bmjopen.bmj.com/site/about/guidelines.xhtml

to the perceived risk of COVID infection.[43] There is therefore a possibility that patients will choose not to undergo any elective procedures in the current climate, nor travel to an unknown hospital for that care. Pooled waiting lists are often disliked by surgeons who site the lack of autonomy and patient ownership with an increased risk of mis-diagnosis, unnecessary procedures listed, and unaddressed patient complexities.[44,45] These risks can, and should, be mitigated by ensuring clear standardised patient pathways, patient triage and suitability assessments, clarity in the named responsible surgeon and pathways for ongoing continuity of care. Virtual platforms have become increasingly available during COVID allowing remote consultation and triaging of patients prior to any procedures.[46]

This study included procedures of varying complexity and ability to increase surge capacity to overcome increased elective waiting lists. Many of the most common procedures featured, including gastrointestinal endoscopy, excisions of skin lesions and joint injection or aspiration may be performed as ‘day case’ procedures and the ability to increase procedural throughput is less encumbered by the need for close anaesthetic support or high dependency recovery space. In comparison, many higher-risk procedures, including complex cardiac, cancer and orthopaedic surgery, are of lower volume. For example, during the study period, in England and Wales 16,000 planned colorectal cancer resections were performed, while 14,500 planned coronary artery bypass graft procedures were performed across the UK.[47,48] These procedures are more likely to require significant anaesthetic support, post-operative critical or high-dependency care and lead to longer inpatient stays. Planning to retain capacity for these complex procedures may therefore entail a different approach to the pooled list approach suggested for the HVPs identified in this study.

Additionally, in using historical surgical volume as a means of quantifying maximum capacity, the study does not incorporate measures to increase surge capacity above prior maximal volumes. As such, the maximal capacities identified for pooled list communities in this study may significantly underestimate the throughput which may be achieved with additional measures to support expansion of surgical capacity.

For peer review only - http://bmjopen.bmj.com/site/about/guidelines.xhtml

For peer review only

Finally, while we have identified a mismatch between current policy (STP boundaries) and practice (the natural networks of surgical providers), we appreciate that implementation of new integrated networks on a larger scale would require significant new resources and planning. A new system of funding flows, mechanisms for regional waiting list coordination and a cost per case mechanism or other financial incentive would be required to support this new model.

The NHS, despite being centrally funded, functions as a disparate collection of separate providers with their own priorities and resource constraints. In the COVID-19 pandemic, pre-existing structures of service delivery within the NHS were temporarily transformed. Primary care providers collaborated at a regional level to provide COVID care through a network of hubs while hospitals collaborated with one another to ensure some cancer care could continue at a smaller number of 'clean' hospital sites. As health systems across the world look to address an ever-growing backlog for planned care created by COVID-19, this trend of enhanced collaboration must continue. If the NHS is to overcome this backlog and cope with further waves of COVID, providers of surgical care must develop the means by which they may share a collective caseload for low-risk patients. What is certain is that the NHS, along with most other healthcare delivery systems, is having to make seismic changes to the way it works in order to best manage ongoing complexities. This study provides a solution with greater regional capacity flexibility with which to respond and adapt. Re-designing arbitrary geographical boundaries to follow expanded natural surgical community networks has the potential to increase efficiency by flexing existing supply to meet demand. This, in addition to other key strategies, could have a profound effect on tackling the massive backlog of cases accruing during this deadly pandemic, thereby preventing further death, disability and reduced productivity from delayed surgery.

For peer review only - http://bmjopen.bmj.com/site/about/guidelines.xhtml

Author Contributions

JC and AM were involved in all aspects of the study. MB was involved in the development of the methodology and assisted in the formal analysis. SM, MB and JK were involved in the planning, conduct and reporting of the study. JC has had access to all the data in the study and all authors had final responsibility for the decision to submit for publication. JC attests that all listed authors meet authorship criteria and that no others meeting the criteria have been omitted.

Funding

This article is independent research supported by grants from The Peter Sowerby Foundation and the National Institute for Health Research (NIHR) Imperial Patient Safety and Translational Research Centre (PSTRC) PSTRC_2016_004. Infrastructure support for this work was provided by the NIHR Imperial Biomedical Research Centre (BRC) 1215-20013. MB and JC acknowledge support from EPSRC grant EP/N014529/1 supporting the EPSRC Centre for Mathematics of Precision Healthcare. JC acknowledges support from the Wellcome Trust [215938/Z/19/Z]. JK reports funding from H2020 - ITN grant, NIHR - i4i and CRUK. Johnson and Johnson have supported the activities of the Pansurg collaborative with an educational grant.

Competing interests

JK reports consultancy for Verb robotics / Ethicon and is a shareholder of One Welbeck Day Surgery.

Acknowledgements

Data management was provided by the Big Data and Analytical Unit (BDAU) at the Institute of Global Health Innovation (IGHI), Imperial College London. The views expressed in this publication are those of the author(s) and not necessarily those of the NHS, the National Institute for Health Research or the Department of Health.

Dissemination declaration

For peer review only - http://bmjopen.bmj.com/site/about/guidelines.xhtml

Data as to hospital allocation to putative surgical communities will be made publicly available on www.healthdatascience.co.uk and will be disseminated to local care providers and commissioners.

Data Sharing Statement

Data used in this study were obtained from NHS Digital for the purpose of this work and may only be accessed through direct application to NHS Digital. Patient-level data is required for the analyses conducted, and therefore sharing of data pertaining to this study is not possible. Data for surgical community assignments are available from the authors on request.

Transparency declaration

The lead author affirms that this manuscript is an honest, accurate, and transparent account of the study being reported; that no important aspects of the study have been omitted; and that any discrepancies from the study as planned have been explained.

Statement of Ethical Approval

This study received local ethical approval through the Imperial College Research Ethics Committee (17IC4178).

For peer review only

For peer review only - http://bmjopen.bmj.com/site/about/guidelines.xhtml

References

1. K. Turaga SG. Are we harming cancer patients by delaying their cancer surgery during the COVID-19 pandemic? *Annals of Surgery* 2020.
2. Mary E Brindle GD, Keith Lillemoe, Atul Gawande. Approaching surgical triage during the COVID-19 Pandemic. *Annals of Surgery* 2020.
3. Surgeons ACo. COVID-19: Elective Case Triage Guidelines for Surgical Care. https://www.facs.org/covid-19/clinical-guidance/elective-case. Published 2020, March 24. Accessed.
4. Surgeons RCo. *COVID-19: Good Practice for Surgeons and Surgical Teams.* Royal College of Surgeons, England 2020.
5. Nepogodiev D, Bhangu A. Elective surgery cancellations due to the COVID-19 pandemic: global predictive modelling to inform surgical recovery plans. *The British journal of surgery.* 2020.
6. Brennan S. 'Mountain' waiting list will need five-year plan, says royal college. In. Vol 2020. Health Services Journal2020.
7. Brennan S. *Trusts told to create 'covid-free hubs' for cancer patients.* Health Services Journal 2020.
8. Kasivisvanathan V, Lindsay J, Rakhshani-moghadam S, et al. Evaluation of 30-day mortality for 500 patients undergoing non-emergency surgery in a COVID-19 cold site within a multicentre regional surgical network during the COVID-19 pandemic. *medRxiv.* 2020:2020.2006.2010.20115543.
9. Office NA. NHS waiting times for elective and cancer treatment. In: England DoHSCaN, ed2019.
10. General CaA. *NHS waiting times for elective and cancer treatment.* Department of Health & Social Care and NHS England;March 2019.
11. Challen R T-AK, Pitt M et al. Estimates of regional infectivity of COVID-19 in the United Kingdom following imposition of social distancing measures. *medRxiv.* 2020.
12. Dixon A RR, Appleby J, Burge P, Devlin N, Magee H. *Patient Choice. How Patients Choose and How Providers Respond. .* The King's Fund;2010.
13. Dawson D, Gravelle H, Jacobs R, Martin S, Smith PC. The effects of expanding patient choice of provider on waiting times: evidence from a policy experiment. *Health Econ.* 2007;16(2):113-128.
14. Service TaCD. *National Clinical Coding Standards OPCS-4. Accurate data for quality information* Health and Social Care Information Centre2019.
15. Quan H, Li B, Couris CM, et al. Updating and validating the Charlson comorbidity index and score for risk adjustment in hospital discharge abstracts using data from 6 countries. *Am J Epidemiol.* 2011;173(6):676-682.
16. Digital N. Estates Returns Information Collection. In: Collection S, ed. NHS Digital 2020.
17. Sinnott RW. Virtues of the Harversine *Sky and Telescope* 1984;68(2):158.
18. Statistics OfN. Lower Layer Super Output Areas (December 2011) Population Weighted Centroids. https://geoportal.statistics.gov.uk/datasets/lower-layer-super-output-areas-december-2011-population-weighted-centroids. Published 2019. Accessed.

For peer review only - http://bmjopen.bmj.com/site/about/guidelines.xhtml

19. Clarke JM, Barahona M, Darzi AW. Defining Hospital Catchment Areas Using Multiscale Community Detection: A Case Study for Planned Orthopaedic Care in England. *bioRxiv.* 2019:619692.
20. Beguerisse-Díaz M, Vangelov B, Barahona M. Finding role communities in directed networks using Role-Based Similarity, Markov Stability and the Relaxed Minimum Spanning Tree. Paper presented at: 2013 IEEE Global Conference on Signal and Information Processing; 3-5 Dec. 2013, 2013.
21. Altuncu MT, Mayer E, Yaliraki SN, Barahona M. From free text to clusters of content in health records: an unsupervised graph partitioning approach. *Applied Network Science.* 2019;4(1):2.
22. Schaub MT, Delvenne J-C, Yaliraki SN, Barahona M. Markov dynamics as a zooming lens for multiscale community detection: non clique-like communities and the field-of-view limit. *PloS one.* 2012;7(2):e32210-e32210.
23. Lambiotte R, Delvenne J, Barahona M. Random Walks, Markov Processes and the Multiscale Modular Organization of Complex Networks. *IEEE Transactions on Network Science and Engineering.* 2014;1(2):76-90.
24. Rhoades SA. Market share inequality, the HHI, and other measures of the firm-composition of a market. *Review of Industrial Organization.* 1995;10(6):657-674.
25. Martin S, Rice N, Jacobs R, Smith P. The market for elective surgery: joint estimation of supply and demand. *J Health Econ.* 2007;26(2):263-285.
26. England N. Advice on maintaining cancer treatment during the COVID-19 response. In: Improvement NEaN, ed2020.
27. PanSurg. Exclusive: The three vital lessons Italian hospitals have learned in fighting covid-19. In: Journal HS, ed. Vol 2020. Health Services Journal2020.
28. Ballini L, Negro A, Maltoni S, et al. Interventions to reduce waiting times for elective procedures. *Cochrane Database of Systematic Reviews.* 2015(2).
29. Leach P, Rutherford SA, King AT, Leggate JR. Generic waiting lists for routine spinal surgery. *Journal of the Royal Society of Medicine.* 2004;97(3):119-120.
30. John Appleby AH, Nancy Devlin. *What is the Real Cost of More Patient Choice?* : The King's Fund;2003.
31. Madsen M, Asmundsson P, Brekke IB, et al. Scandiatransplant: thirty years of cooperation in organ transplantation in the Nordic countries. *Clin Transpl.* 1998:121-131.
32. Siciliani L, Martin S. An empirical analysis of the impact of choice on waiting times. *Health Econ.* 2007;16(8):763-779.
33. Dawson D, Jacobs R, Martin S, Smith P. Is patient choice an effective mechanism to reduce waiting times? *Appl Health Econ Health Policy.* 2004;3(4):195-203.
34. Dixon A. *New Labour's Market Reforms - Patient Choice of Hospital.* 2011.
35. Damiani M, Propper C, Dixon J. Mapping choice in the NHS: cross sectional study of routinely collected data. *BMJ (Clinical research ed).* 2005;330(7486):284.
36. Ryan M, McIntosh E, Dean T, Old P. Trade-offs between location and waiting times in the provision of health care: the case of elective surgery on the Isle of Wight. *J Public Health Med.* 2000;22(2):202-210.

For peer review only - http://bmjopen.bmj.com/site/about/guidelines.xhtml

37. Aggarwal A, Lewis D, Mason M, Purushotham A, Sullivan R, van der Meulen J. Effect of patient choice and hospital competition on service configuration and technology adoption within cancer surgery: a national, population-based study. *Lancet Oncol.* 2017;18(11):1445-1453.
38. Pandit JJ, Pandit M, Reynard JM. Understanding waiting lists as the matching of surgical capacity to demand: are we wasting enough surgical time? *Anaesthesia.* 2010;65(6):625-640.
39. John Appleby VR, Francesca Frosini, Gwyn Bevan, Haiyan Gao, Tom Lyscom. Variations in Health Care. The King's Fund. https://www.kingsfund.org.uk/sites/default/files/field/field_publication_file/Variations-in-health-care-good-bad-inexplicable-report-The-Kings-Fund-April-2011.pdf. Published 2011. Accessed August, 2020.
40. Markar SR, Clarke J, Kinross J. Practice patterns of diagnostic upper gastrointestinal endoscopy during the initial COVID-19 outbreak in England. *Lancet Gastroenterol Hepatol.* 2020;5(9):804-805.
41. Permain N. COVID-19: Update on partnership working with the Independent Sector Providers and the Independent Healthcare Providers Network (IHPN) In: Teams RDRL, edAugust 2020.
42. The Health Protection (Coronavirus, Restrictions) (England) Regulations 2020. In:2020.
43. Owens B. Reduced attendances and admissions: lessons from the covid crisis. In: Journal HS, ed. Vol 2020: HSJ; 2020.
44. Ramchandani M, Mirza S, Sharma A, Kirkby G. Pooled cataract waiting lists: views of hospital consultants, general practitioners and patients. *Journal of the Royal Society of Medicine.* 2002;95(12):598-600.
45. Position Statement on Pooled Waiting Lists [press release]. British Orthopaedic Association2013.
46. England N. Elective Care Transformation Programme In: England N, ed2020.
47. National Institute for Cardiovascular Outcomes Research. *NATIONAL ADULT CARDIAC SURGERY AUDIT, 2019 SUMMARY REPORT (2015/16-2017/18 DATA).* 2019.
48. National Bowel Cancer Audit. *National Bowel Cancer Audit, Annual Report 2019. An audit of the care received by people with Bowel Cancer in England and Wales.* 2019.

For peer review only

For peer review only - http://bmjopen.bmj.com/site/about/guidelines.xhtml

Figure captions:

Figure 1: Division of England into 45, 16 and 7 surgical communities (in colour). according to Markov Stability. STP boundaries are overlaid (black lines).

Figure 2: The equivalent number of surgical communities active in each STP as determined by the EMS. Areas of darker blue, 4 (e.g. East Anglia), represent those areas with greatest difference between surgical communities and STPs, whereas lighter blue shows greater agreement (e.g. Cornwall).

Figure 3: The absolute percentage difference between the number of patients undergoing surgery resident within a surgical community (demand) and the number of procedures performed by hospitals within the community (supply). Areas in blue represent those surgical communities where procedures performed on patients outnumber those performed by the local providers.

For peer review only

For peer review only - http://bmjopen.bmj.com/site/about/guidelines.xhtml

		Charlson Score		
		0	1-2	3+
**Age**	< 60	Low	Low	Medium
	60 - 74	Low	Medium	High
	75+	Medium	High	High

Table 1: Classification of low, medium and high-risk patients based on age and Charlson score.

For peer review only

For peer review only - http://bmjopen.bmj.com/site/about/guidelines.xhtml

		Patient risk			
Procedure	Total no. of cases	Low risk (%)	Medium risk (%)	High risk (%)	Mean distance travelled, Kms
Lower GI endoscopy	937,616	74.8	17.9	7.3	9.8
Upper GI endoscopy	650,133	66.9	22.1	10.9	9.4
Lens extraction + replacement	395,445	33.5	46.5	20.0	10.9
Excision of skin lesion	215,608	55.0	29.5	15.5	12.7
Injection/aspiration joint	142,562	71.6	20.9	7.5	12.6
Vitrectomy	132,938	39.9	44.1	16.1	13.2
Cystoscopy	130,114	56.4	26.2	17.4	11.8
Insertion central venous catheter	109,864	24.3	38.3	37.4	14.0
Coronary angiography	105,620	56.2	30.0	13.8	13.9
Dental extraction	101,435	91.6	5.8	2.5	11.5
Knee replacement	78,773	53.3	34.4	12.3	13.4
Bladder catheterisation or irrigation	71,552	42.5	32.7	24.8	12.8
Injection to bladder	67,167	34.3	29.8	35.9	11.5
Spinal facet joint injection	64,154	70.4	21.9	7.7	14.0
Cholecystectomy	61,790	80.5	13.8	5.7	11.8
Lymph node biopsy	60,674	34.8	34.4	30.8	14.9
Epidural or spinal injection	60,656	69.2	22.6	8.1	12.9
Inguinal hernia repair	58,943	72.6	19.9	7.5	10.9
Spinal nerve root injection	58,212	77.0	17.5	5.5	16.2
Knee meniscectomy/ meniscal repair	57,871	93.2	5.9	0.8	12.4
Hysteroscopy	52,360	90.9	6.4	2.7	9.9
Carpal tunnel release	48,245	70.7	22.2	7.1	11.0
Application/ removal of internal fixation of bone	46,771	84.6	11.9	3.5	15.7
Dental clearance	43,463	82.3	11.7	5.9	11.1
Partial breast excision	41,827	50.1	31.4	18.5	11.5
Bone marrow biopsy	38,369	39.8	34.8	25.5	15.6
Primary joint resurfacing	37,854	59.3	30.1	10.5	14.0
Cystoscopy + resection of bladder lesion	37,458	17.7	29.8	52.5	11.2

Table 2: The 28 procedures accounting for more than half of all elective surgical activity in England. The proportion of patients classified as low, medium and high risk according to Table 1 are shown, along with the mean distance travelled from a patient's LSOA of residence to the hospital site in which the procedure is performed.

For peer review only - http://bmjopen.bmj.com/site/about/guidelines.xhtml

Partition	A	B	C
Number of communities	45	16	7
Median number of cases per community	78998 (43628 - 118087)	214216 (122823 - 314022)	574403 (406465 - 679703)
Median number of treatment sites per community	9 (5 - 17)	25 (19 - 44)	84 (56 - 98)
Absolute supply:demand mismatch (%)	5.1 (2.9 - 10.2)	4.1 (1.0 - 5.7)	2.2 (1.0 - 2.9)

Table 3: Descriptive statistics for the three optimal partitions produced. Interquartile ranges are shown in parentheses where appropriate.

For peer review only

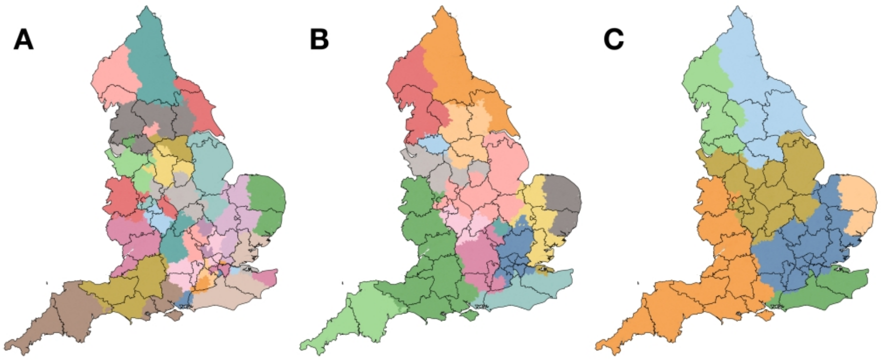

Figure 1
Division of England into 45, 16 and 7 surgical communities (in colour). according to Markov Stability. STP boundaries are overlaid (black lines).

338x190mm (300 x 300 DPI)

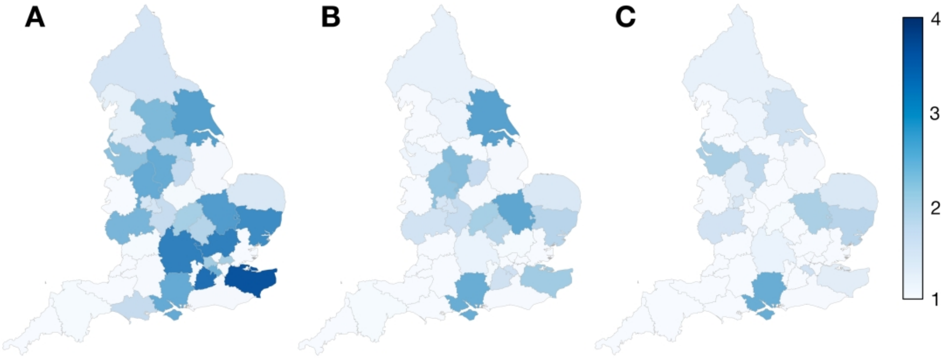

Figure 2
The equivalent number of surgical communities active in each STP as determined by the EMS. Areas of darker blue, 4 (e.g. East Anglia), represent those areas with greatest difference between surgical communities and STPs, whereas lighter blue shows greater agreement (e.g. Cornwall).

338x190mm (300 x 300 DPI)

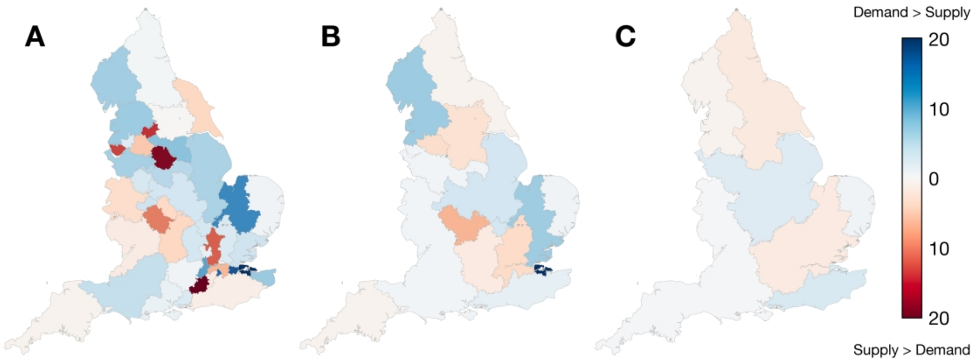

Figure 3
The absolute percentage difference between the number of patients undergoing surgery resident within a surgical community (demand) and the number of procedures performed by hospitals within the community (supply). Areas in blue represent those surgical communities where procedures performed on patients outnumber those performed by the local providers.

338x190mm (300 x 300 DPI)

Appendix

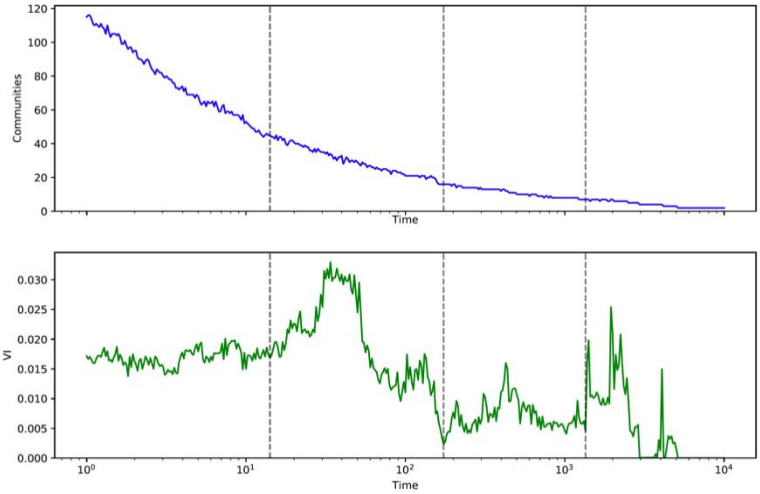

Supplemental Figure 1: Markov Multiscale Community Detection output showing the number of communities in the optimal network partition (top) and variation of information between partitions produced for Markov times from 1 to 10,000. Vertical lines indicate the three partitions of surgical communities selected for further review.

For peer review only

The RECORD statement – checklist of items, extended from the STROBE statement, that should be reported in observational studies using routinely collected health data.

	Item No.	STROBE items	Location in manuscript where items are reported	RECORD items	Location in manuscript where items are reported
**Title and abstract**					
	1	(a) Indicate the study's design with a commonly used term in the title or the abstract (b) Provide in the abstract an informative and balanced summary of what was done and what was found	Abstract and title (pages 1 and 3)	RECORD 1.1: The type of data used should be specified in the title or abstract. When possible, the name of the databases used should be included.<br><br>RECORD 1.2: If applicable, the geographic region and timeframe within which the study took place should be reported in the title or abstract.<br><br>RECORD 1.3: If linkage between databases was conducted for the study, this should be clearly stated in the title or abstract.	Abstract (page 3)<br><br>Abstract (page 3)<br><br>N/A
**Introduction**					
Background rationale	2	Explain the scientific background and rationale for the investigation being reported	Introduction (pages 5 and 6)		
Objectives	3	State specific objectives, including any prespecified hypotheses	Introduction (page 6)		
**Methods**					
Study Design	4	Present key elements of study design early in the paper	Methods (pages 6 and 7)		
Setting	5	Describe the setting, locations, and relevant dates, including periods of recruitment, exposure, follow-up, and data collection	Methods (pages 6 and 7)		

For peer review only

Participants	6	*(a) Cohort study* - Give the eligibility criteria, and the sources and methods of selection of participants. Describe methods of follow-up<br>*Case-control study* - Give the eligibility criteria, and the sources and methods of case ascertainment and control selection. Give the rationale for the choice of cases and controls<br>*Cross-sectional study* - Give the eligibility criteria, and the sources and methods of selection of participants<br><br>*(b) Cohort study* - For matched studies, give matching criteria and number of exposed and unexposed<br>*Case-control study* - For matched studies, give matching criteria and the number of controls per case	Methods (pages 6 and 7)	RECORD 6.1: The methods of study population selection (such as codes or algorithms used to identify subjects) should be listed in detail. If this is not possible, an explanation should be provided.<br><br>RECORD 6.2: Any validation studies of the codes or algorithms used to select the population should be referenced. If validation was conducted for this study and not published elsewhere, detailed methods and results should be provided.<br><br>RECORD 6.3: If the study involved linkage of databases, consider use of a flow diagram or other graphical display to demonstrate the data linkage process, including the number of individuals with linked data at each stage.	Methods (pages 6 and 7)<br><br>Methods (pages 6 and 7)<br><br>N/A
Variables	7	Clearly define all outcomes, exposures, predictors, potential confounders, and effect modifiers. Give diagnostic criteria, if applicable.	Methods (page 7)	RECORD 7.1: A complete list of codes and algorithms used to classify exposures, outcomes, confounders, and effect modifiers should be provided. If these cannot be reported, an explanation should be provided.	N/A
Data sources/ measurement	8	For each variable of interest, give sources of data and details of methods of assessment (measurement).<br>Describe comparability of assessment methods if there is more than one group	Methods (page 7)		

For peer review only

Bias	9	Describe any efforts to address potential sources of bias	Methods (pages 6, 7 and 8)		
Study size	10	Explain how the study size was arrived at	Methods (page 6)		
Quantitative variables	11	Explain how quantitative variables were handled in the analyses. If applicable, describe which groupings were chosen, and why	Methods (pages 7, 8 and 9)		
Statistical methods	12	(a) Describe all statistical methods, including those used to control for confounding<br>(b) Describe any methods used to examine subgroups and interactions<br>(c) Explain how missing data were addressed<br>(d) *Cohort study* - If applicable, explain how loss to follow-up was addressed<br>*Case-control study* - If applicable, explain how matching of cases and controls was addressed<br>*Cross-sectional study* - If applicable, describe analytical methods taking account of sampling strategy<br>(e) Describe any sensitivity analyses	Methods (pages 7, 8 and 9)		
Data access and cleaning methods		..		RECORD 12.1: Authors should describe the extent to which the investigators had access to the database population used to create the study population.	Methods (pages 6 and 7)

For peer review only

				RECORD 12.2: Authors should provide information on the data cleaning methods used in the study.	Methods (pages 6 and 7)
Linkage		..		RECORD 12.3: State whether the study included person-level, institutional-level, or other data linkage across two or more databases. The methods of linkage and methods of linkage quality evaluation should be provided.	N/A
**Results**					
Participants	13	(a) Report the numbers of individuals at each stage of the study (*e.g.*, numbers potentially eligible, examined for eligibility, confirmed eligible, included in the study, completing follow-up, and analysed)<br>(b) Give reasons for non-participation at each stage.<br>(c) Consider use of a flow diagram	Results (page 10)	RECORD 13.1: Describe in detail the selection of the persons included in the study (*i.e.,* study population selection) including filtering based on data quality, data availability and linkage. The selection of included persons can be described in the text and/or by means of the study flow diagram.	Methods (pages 6 and 7)
Descriptive data	14	(a) Give characteristics of study participants (*e.g.*, demographic, clinical, social) and information on exposures and potential confounders<br>(b) Indicate the number of participants with missing data for each variable of interest<br>(c) *Cohort study* - summarise follow-up time (*e.g.*, average and total amount)	Results (pages 10 and 11)		
Outcome data	15	*Cohort study* - Report numbers of outcome events or summary measures over time<br>*Case-control study* - Report numbers in each exposure			

For peer review only

		category, or summary measures of exposure<br>*Cross-sectional study* - Report numbers of outcome events or summary measures	Results (pages 10 and 11)		
Main results	16	(a) Give unadjusted estimates and, if applicable, confounder-adjusted estimates and their precision (e.g., 95% confidence interval). Make clear which confounders were adjusted for and why they were included<br>(b) Report category boundaries when continuous variables were categorized<br>(c) If relevant, consider translating estimates of relative risk into absolute risk for a meaningful time period	Results (pages 10 and 11)		
Other analyses	17	Report other analyses done—e.g., analyses of subgroups and interactions, and sensitivity analyses	Results (pages 10 and 11)		
**Discussion**					
Key results	18	Summarise key results with reference to study objectives	Discussion (page 12)		
Limitations	19	Discuss limitations of the study, taking into account sources of potential bias or imprecision. Discuss both direction and magnitude of any potential bias	Discussion (page 14)	RECORD 19.1: Discuss the implications of using data that were not created or collected to answer the specific research question(s). Include discussion of misclassification bias, unmeasured confounding, missing data, and changing eligibility over time, as they pertain to the study being reported.	Discussion (page 14)
Interpretation	20	Give a cautious overall interpretation of results considering objectives,	Discussion (page 14)		

For peer review only - http://bmjopen.bmj.com/site/about/guidelines.xhtml

		limitations, multiplicity of analyses, results from similar studies, and other relevant evidence			
Generalisability	21	Discuss the generalisability (external validity) of the study results	Discussion (pages 12, 13 and 14)		
**Other Information**					
Funding	22	Give the source of funding and the role of the funders for the present study and, if applicable, for the original study on which the present article is based	Page 16		
Accessibility of protocol, raw data, and programming code		..		RECORD 22.1: Authors should provide information on how to access any supplemental information such as the study protocol, raw data, or programming code.	Page 17

*Reference: Benchimol EI, Smeeth L, Guttmann A, Harron K, Moher D, Petersen I, Sørensen HT, von Elm E, Langan SM, the RECORD Working Committee. The REporting of studies Conducted using Observational Routinely-collected health Data (RECORD) Statement. *PLoS Medicine* 2015; in press.

*Checklist is protected under Creative Commons Attribution (CC BY) license.

For peer review only
